# Supplementary material for: Injury-induced perivascular niche supports alternative differentiation of adult rodent CNS progenitor cells
Source: eLife. 2018 Sep 17;7:e30325. doi: 10.7554/eLife.30325 (PMC6141235; doi:10.7554/eLife.30325)
Supplement: Figure 2—source data 1. — Dots represent level of expression for individual animal sample. [file elife-30325-fig2-data1.pdf]

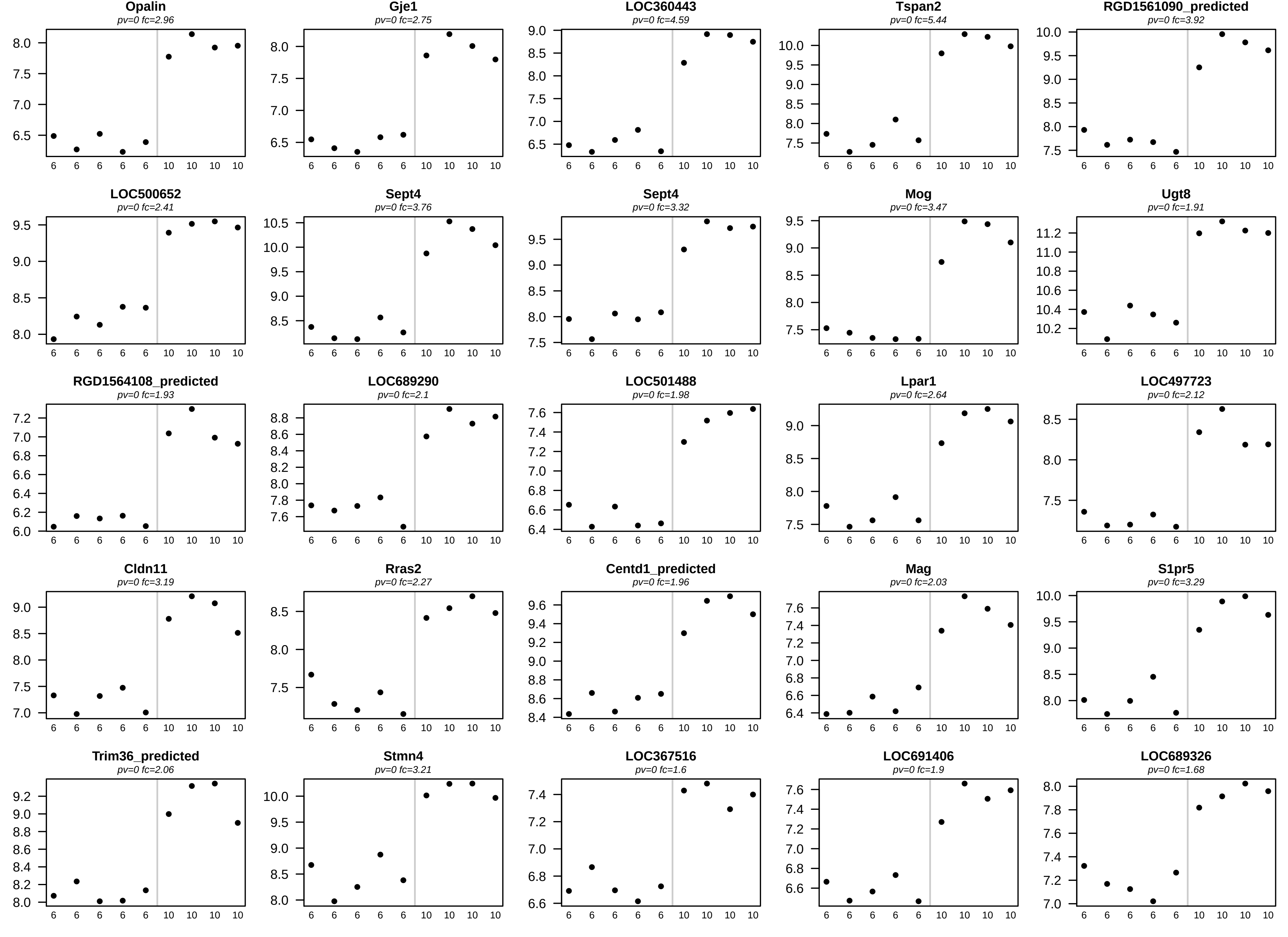
Opalin
pv=0 fc=2.96
Gje1
pv=0 fc=2.75
LOC360443
pv=0 fc=4.59
Tspan2
pv=0 fc=5.44
RGD1561090_predicted
pv=0 fc=3.92
LOC500652
pv=0 fc=2.41
Sept4
pv=0 fc=3.76
Sept4
pv=0 fc=3.32
Mog
pv=0 fc=3.47
Ugt8
pv=0 fc=1.91
RGD1564108_predicted
pv=0 fc=1.93
LOC689290
pv=0 fc=2.1
LOC501488
pv=0 fc=1.98
Lpar1
pv=0 fc=2.64
LOC497723
pv=0 fc=2.12
Cldn11
pv=0 fc=3.19
Rras2
pv=0 fc=2.27
Centd1_predicted
pv=0 fc=1.96
Mag
pv=0 fc=2.03
S1pr5
pv=0 fc=3.29
Trim36_predicted
pv=0 fc=2.06
Stmn4
pv=0 fc=3.21
LOC367516
pv=0 fc=1.6
LOC691406
pv=0 fc=1.9
LOC689326
pv=0 fc=1.68

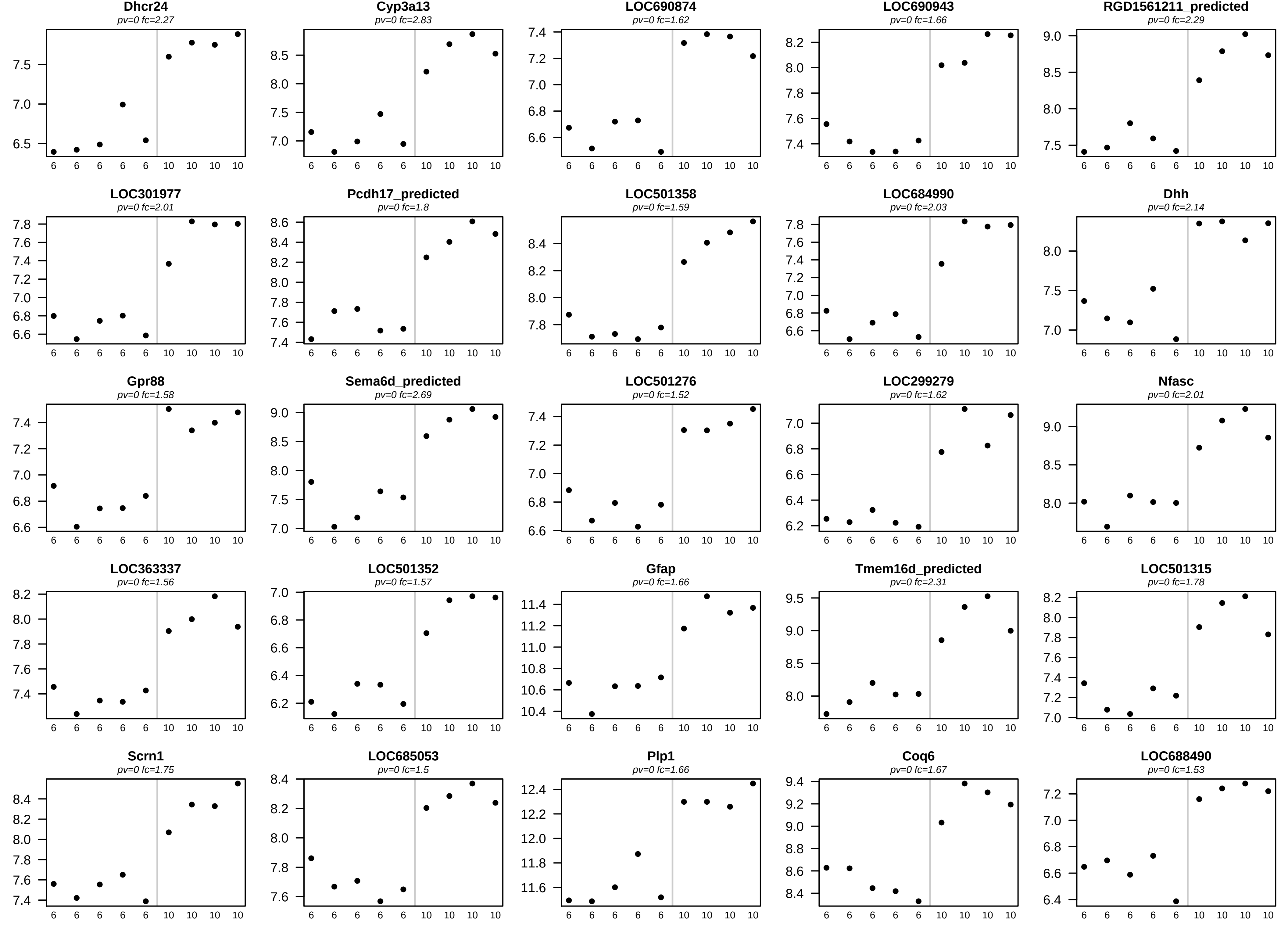
Dhcr24
pv=0 fc=2.27
Cyp3a13
pv=0 fc=2.83
LOC690874
pv=0 fc=1.62
LOC690943
pv=0 fc=1.66
RGD1561211_predicted
pv=0 fc=2.29
LOC301977
pv=0 fc=2.01
Pcdh17_predicted
pv=0 fc=1.8
LOC501358
pv=0 fc=1.59
LOC684990
pv=0 fc=2.03
Dhh
pv=0 fc=2.14
Gpr88
pv=0 fc=1.58
Sema6d_predicted
pv=0 fc=2.69
LOC501276
pv=0 fc=1.52
LOC299279
pv=0 fc=1.62
Nfasc
pv=0 fc=2.01
LOC363337
pv=0 fc=1.56
LOC501352
pv=0 fc=1.57
Gfap
pv=0 fc=1.66
Tmem16d_predicted
pv=0 fc=2.31
LOC501315
pv=0 fc=1.78
Scrn1
pv=0 fc=1.75
LOC685053
pv=0 fc=1.5
Plp1
pv=0 fc=1.66
Coq6
pv=0 fc=1.67
LOC688490
pv=0 fc=1.53

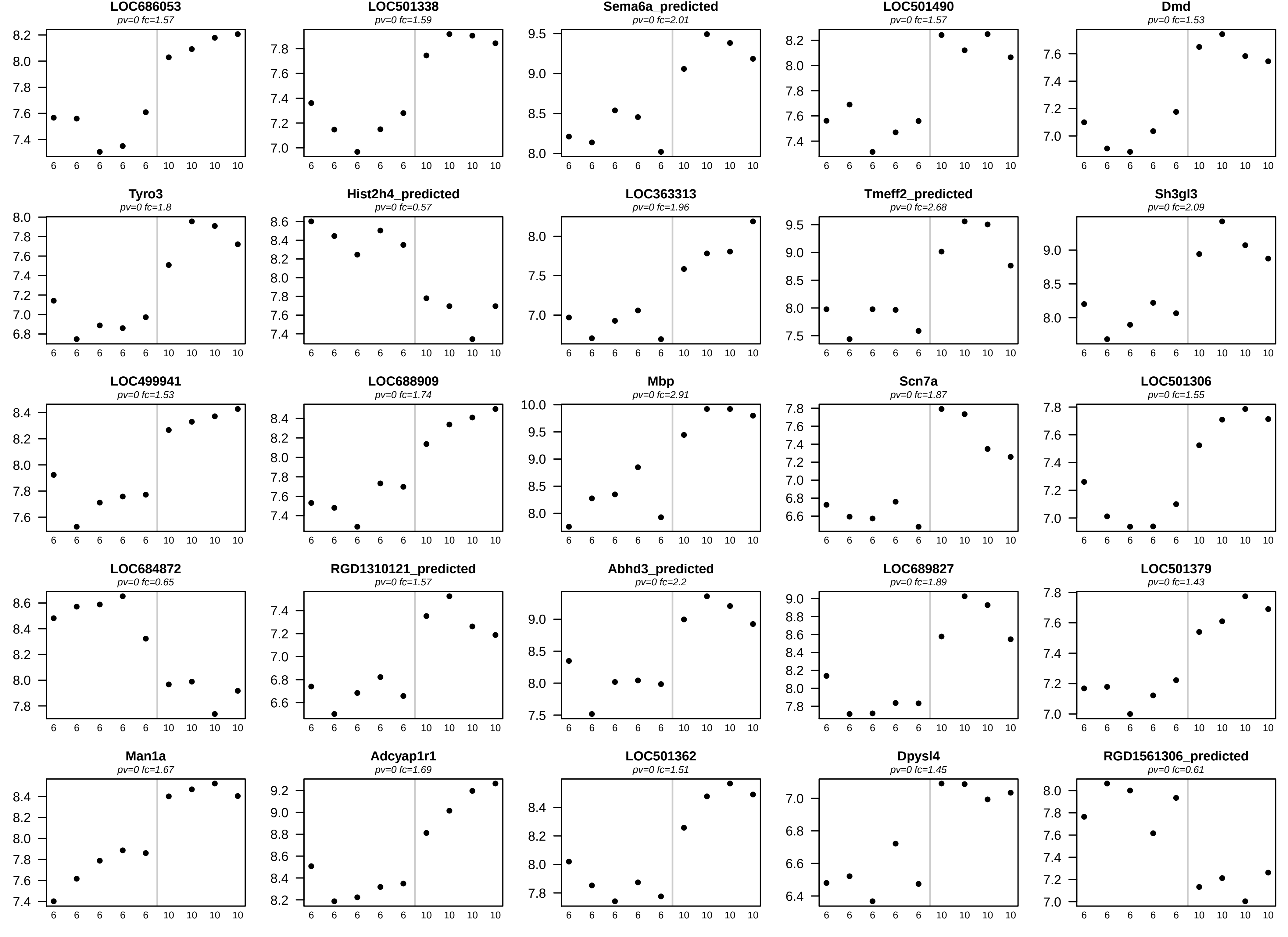
LOC686053
pv=0 fc=1.57
LOC501338
pv=0 fc=1.59
Sema6a_predicted
pv=0 fc=2.01
LOC501490
pv=0 fc=1.57
Dmd
pv=0 fc=1.53
Tyro3
pv=0 fc=1.8
Hist2h4_predicted
pv=0 fc=0.57
LOC363313
pv=0 fc=1.96
Tmeff2_predicted
pv=0 fc=2.68
Sh3gl3
pv=0 fc=2.09
LOC499941
pv=0 fc=1.53
LOC688909
pv=0 fc=1.74
Mbp
pv=0 fc=2.91
Scn7a
pv=0 fc=1.87
LOC501306
pv=0 fc=1.55
LOC684872
pv=0 fc=0.65
RGD1310121_predicted
pv=0 fc=1.57
Abhd3_predicted
pv=0 fc=2.2
LOC689827
pv=0 fc=1.89
LOC501379
pv=0 fc=1.43
Man1a
pv=0 fc=1.67
Adcyap1r1
pv=0 fc=1.69
LOC501362
pv=0 fc=1.51
Dpysl4
pv=0 fc=1.45
RGD1561306_predicted
pv=0 fc=0.61

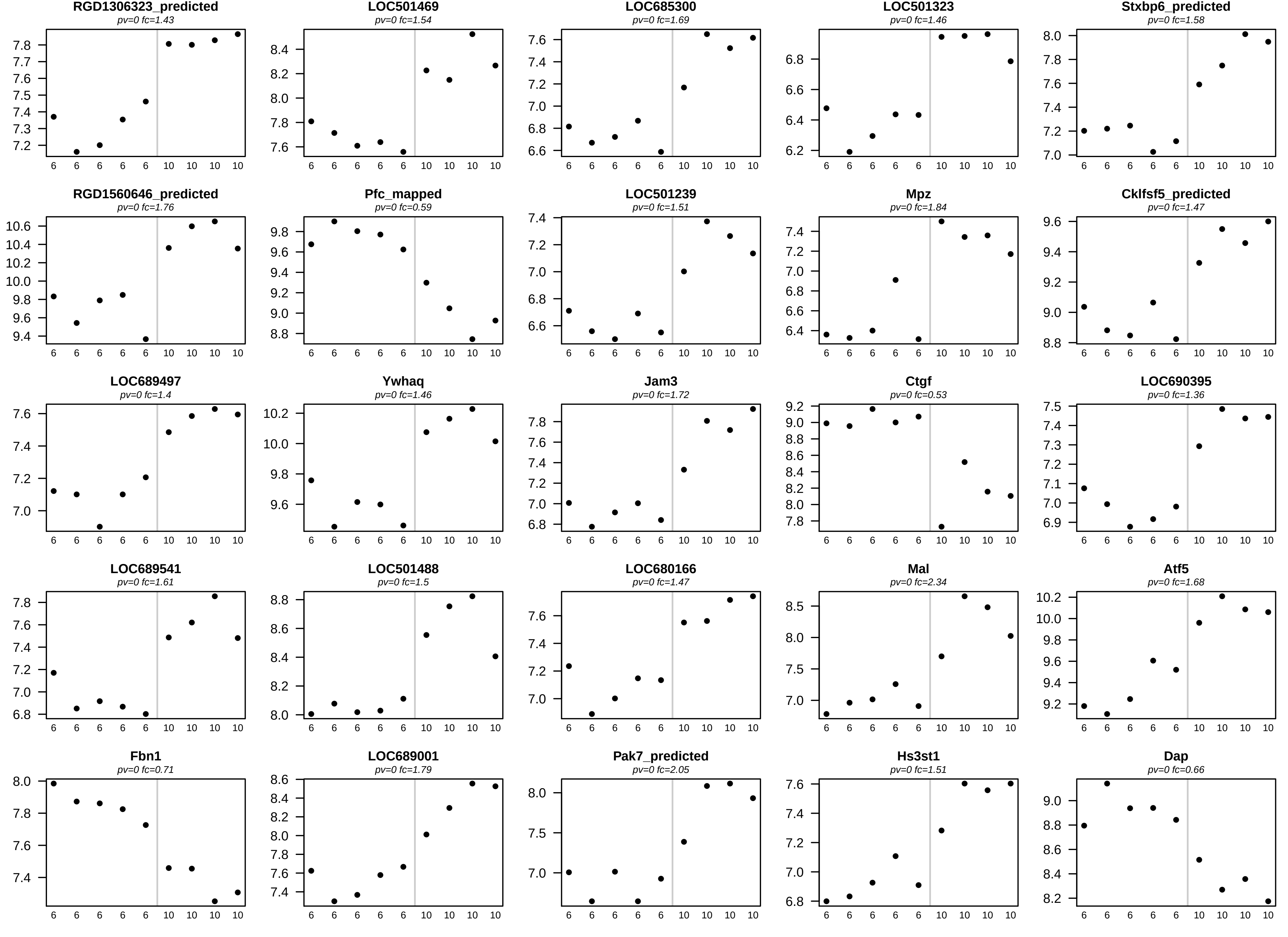
RGD1306323_predicted
pv=0 fc=1.43
LOC501469
pv=0 fc=1.54
LOC685300
pv=0 fc=1.69
LOC501323
pv=0 fc=1.46
Stxbp6_predicted
pv=0 fc=1.58
RGD1560646_predicted
pv=0 fc=1.76
Pfc_mapped
pv=0 fc=0.59
LOC501239
pv=0 fc=1.51
Mpz
pv=0 fc=1.84
Cklfsf5_predicted
pv=0 fc=1.47
LOC689497
pv=0 fc=1.4
Ywhaq
pv=0 fc=1.46
Jam3
pv=0 fc=1.72
Ctgf
pv=0 fc=0.53
LOC690395
pv=0 fc=1.36
LOC689541
pv=0 fc=1.61
LOC501488
pv=0 fc=1.5
LOC680166
pv=0 fc=1.47
Mal
pv=0 fc=2.34
Atf5
pv=0 fc=1.68
Fbn1
pv=0 fc=0.71
LOC689001
pv=0 fc=1.79
Pak7_predicted
pv=0 fc=2.05
Hs3st1
pv=0 fc=1.51
Dap
pv=0 fc=0.66
6 6 6 6 6 10 10 10 10

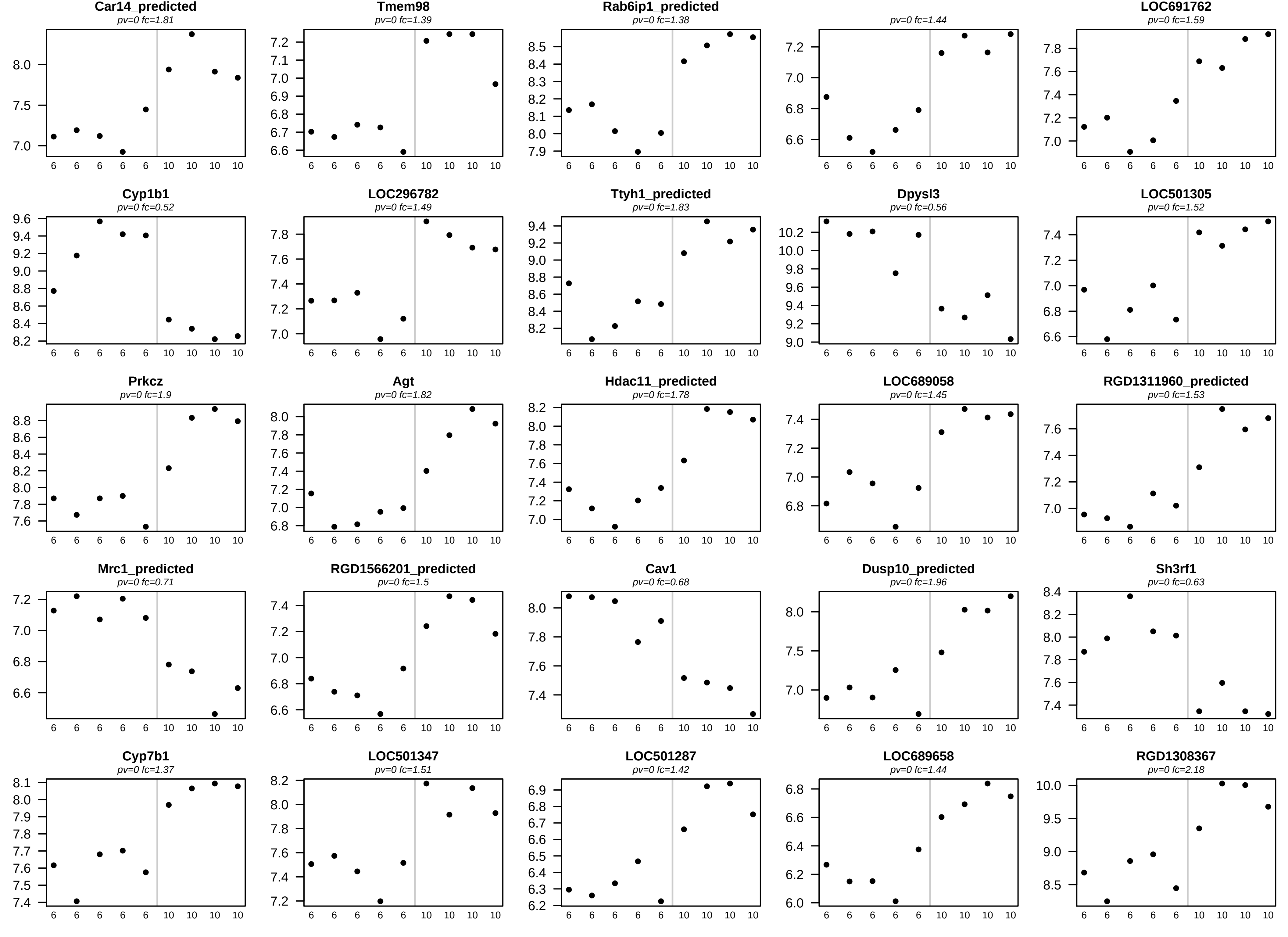
Car14_predicted
pv=0 fc=1.81
Tmem98
pv=0 fc=1.39
Rab6ip1_predicted
pv=0 fc=1.38
pv=0 fc=1.44
LOC691762
pv=0 fc=1.59
Cyp1b1
pv=0 fc=0.52
LOC296782
pv=0 fc=1.49
Ttyh1_predicted
pv=0 fc=1.83
Dpysl3
pv=0 fc=0.56
LOC501305
pv=0 fc=1.52
Prkcz
pv=0 fc=1.9
Agt
pv=0 fc=1.82
Hdac11_predicted
pv=0 fc=1.78
LOC689058
pv=0 fc=1.45
RGD1311960_predicted
pv=0 fc=1.53
Mrc1_predicted
pv=0 fc=0.71
RGD1566201_predicted
pv=0 fc=1.5
Cav1
pv=0 fc=0.68
Dusp10_predicted
pv=0 fc=1.96
Sh3rf1
pv=0 fc=0.63
Cyp7b1
pv=0 fc=1.37
LOC501347
pv=0 fc=1.51
LOC501287
pv=0 fc=1.42
LOC689658
pv=0 fc=1.44
RGD1308367
pv=0 fc=2.18

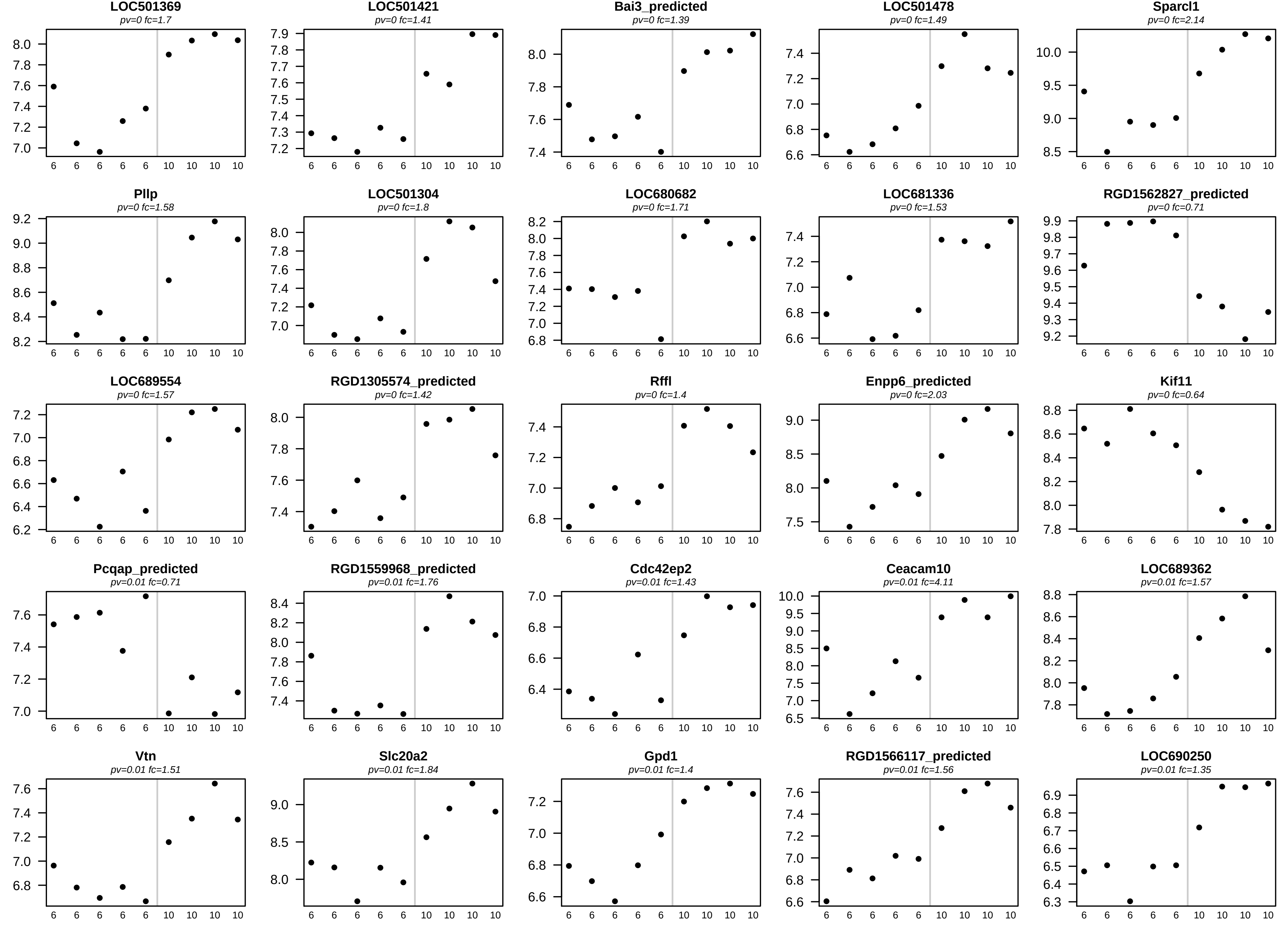
LOC501369
pv=0 fc=1.7
LOC501421
pv=0 fc=1.41
Bai3_predicted
pv=0 fc=1.39
LOC501478
pv=0 fc=1.49
Sparcl1
pv=0 fc=2.14
Pllp
pv=0 fc=1.58
LOC501304
pv=0 fc=1.8
LOC680682
pv=0 fc=1.71
LOC681336
pv=0 fc=1.53
RGD1562827_predicted
pv=0 fc=0.71
LOC689554
pv=0 fc=1.57
RGD1305574_predicted
pv=0 fc=1.42
Rffl
pv=0 fc=1.4
Enpp6_predicted
pv=0 fc=2.03
Kif11
pv=0 fc=0.64
Pcqap_predicted
pv=0.01 fc=0.71
RGD1559968_predicted
pv=0.01 fc=1.76
Cdc42ep2
pv=0.01 fc=1.43
Ceacam10
pv=0.01 fc=4.11
LOC689362
pv=0.01 fc=1.57
Vtn
pv=0.01 fc=1.51
Slc20a2
pv=0.01 fc=1.84
Gpd1
pv=0.01 fc=1.4
RGD1566117_predicted
pv=0.01 fc=1.56
LOC690250
pv=0.01 fc=1.35

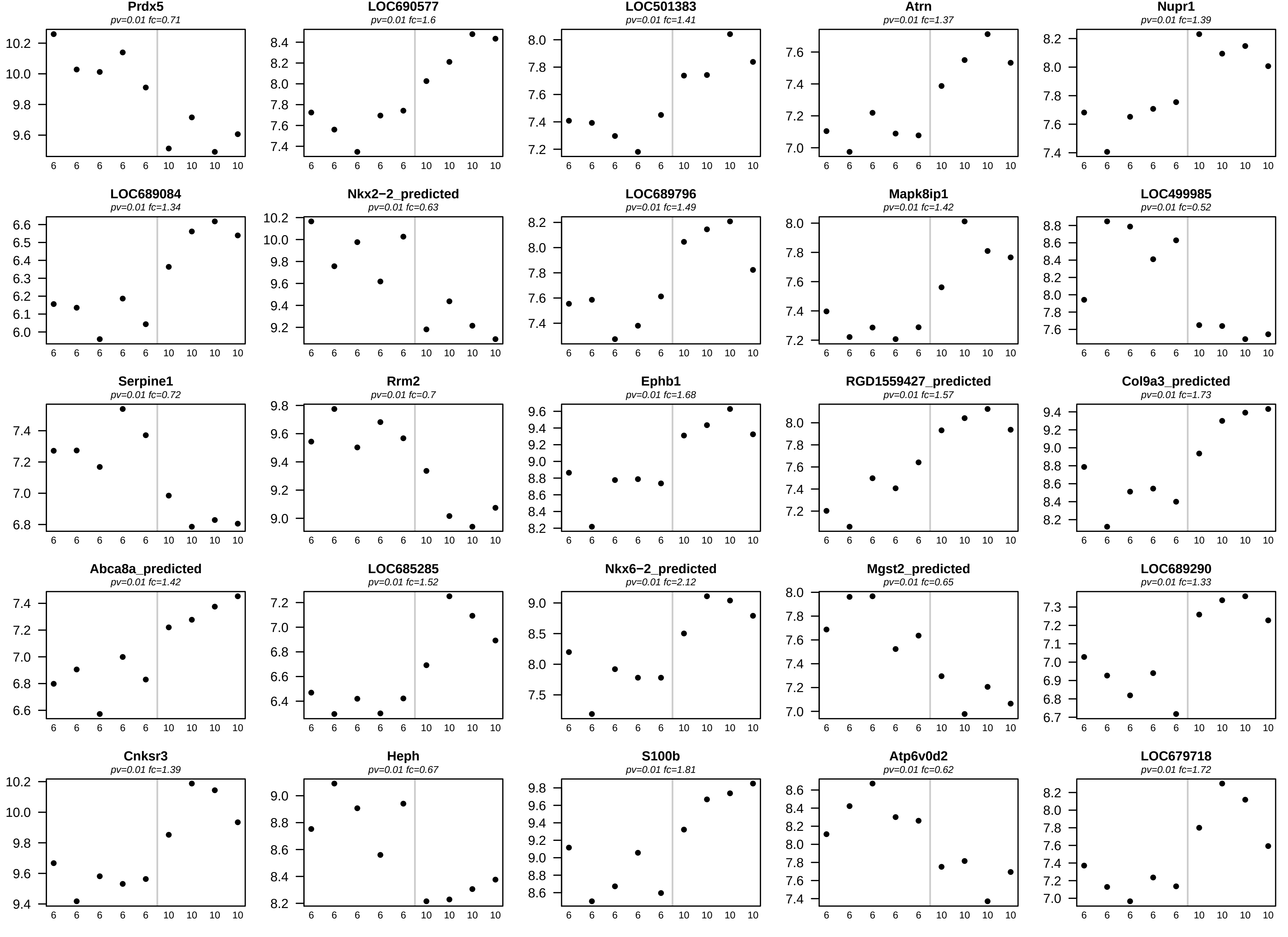
Prdx5
pv=0.01 fc=0.71
LOC690577
pv=0.01 fc=1.6
LOC501383
pv=0.01 fc=1.41
Atrn
pv=0.01 fc=1.37
Nupr1
pv=0.01 fc=1.39
LOC689084
pv=0.01 fc=1.34
Nkx2−2_predicted
pv=0.01 fc=0.63
LOC689796
pv=0.01 fc=1.49
Mapk8ip1
pv=0.01 fc=1.42
LOC499985
pv=0.01 fc=0.52
Serpine1
pv=0.01 fc=0.72
Rrm2
pv=0.01 fc=0.7
Ephb1
pv=0.01 fc=1.68
RGD1559427_predicted
pv=0.01 fc=1.57
Col9a3_predicted
pv=0.01 fc=1.73
Abca8a_predicted
pv=0.01 fc=1.42
LOC685285
pv=0.01 fc=1.52
Nkx6−2_predicted
pv=0.01 fc=2.12
Mgst2_predicted
pv=0.01 fc=0.65
LOC689290
pv=0.01 fc=1.33
Cnksr3
pv=0.01 fc=1.39
Heph
pv=0.01 fc=0.67
S100b
pv=0.01 fc=1.81
Atp6v0d2
pv=0.01 fc=0.62
LOC679718
pv=0.01 fc=1.72

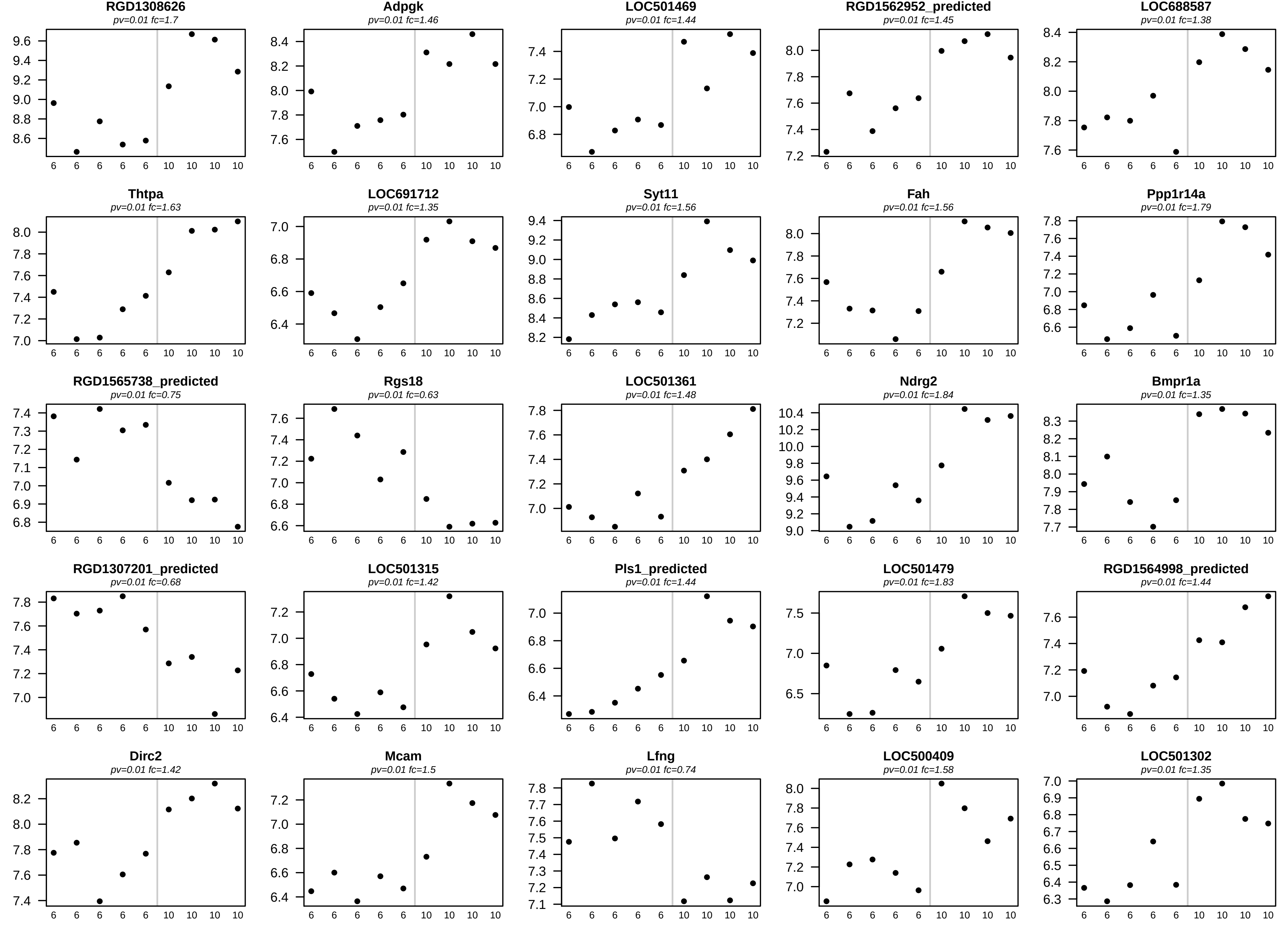
RGD1308626
pv=0.01 fc=1.7
Adpgk
pv=0.01 fc=1.46
LOC501469
pv=0.01 fc=1.44
RGD1562952_predicted
pv=0.01 fc=1.45
LOC688587
pv=0.01 fc=1.38
Thtpa
pv=0.01 fc=1.63
LOC691712
pv=0.01 fc=1.35
Syt11
pv=0.01 fc=1.56
Fah
pv=0.01 fc=1.56
Ppp1r14a
pv=0.01 fc=1.79
RGD1565738_predicted
pv=0.01 fc=0.75
Rgs18
pv=0.01 fc=0.63
LOC501361
pv=0.01 fc=1.48
Ndrg2
pv=0.01 fc=1.84
Bmpr1a
pv=0.01 fc=1.35
RGD1307201_predicted
pv=0.01 fc=0.68
LOC501315
pv=0.01 fc=1.42
Pls1_predicted
pv=0.01 fc=1.44
LOC501479
pv=0.01 fc=1.83
RGD1564998_predicted
pv=0.01 fc=1.44
Dirc2
pv=0.01 fc=1.42
Mcam
pv=0.01 fc=1.5
Lfng
pv=0.01 fc=0.74
LOC500409
pv=0.01 fc=1.58
LOC501302
pv=0.01 fc=1.35

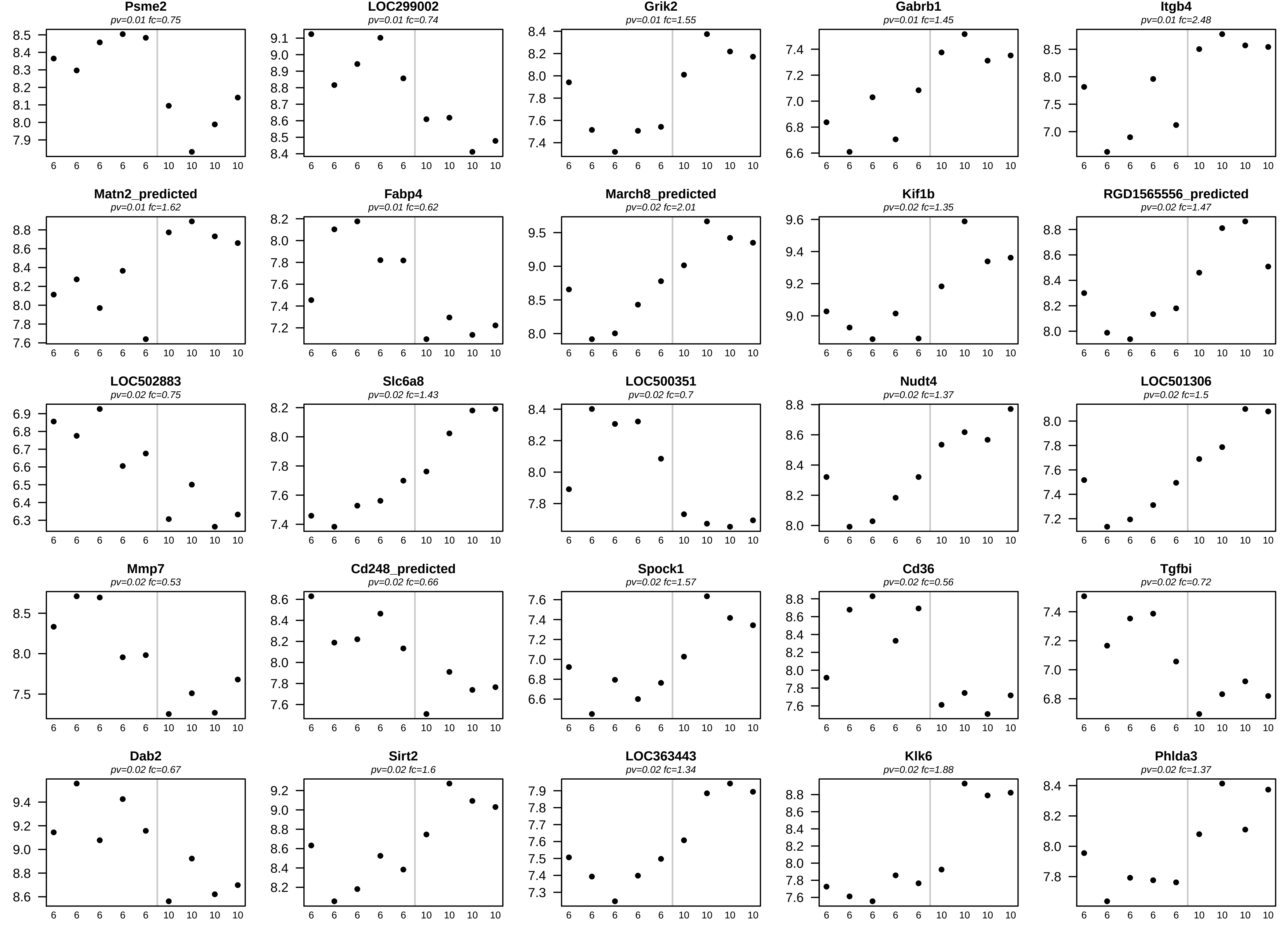
Psme2
pv=0.01 fc=0.75
LOC299002
pv=0.01 fc=0.74
Grik2
pv=0.01 fc=1.55
Gabrb1
pv=0.01 fc=1.45
Itgb4
pv=0.01 fc=2.48
Matn2_predicted
pv=0.01 fc=1.62
Fabp4
pv=0.01 fc=0.62
March8_predicted
pv=0.02 fc=2.01
Kif1b
pv=0.02 fc=1.35
RGD1565556_predicted
pv=0.02 fc=1.47
LOC502883
pv=0.02 fc=0.75
Slc6a8
pv=0.02 fc=1.43
LOC500351
pv=0.02 fc=0.7
Nudt4
pv=0.02 fc=1.37
LOC501306
pv=0.02 fc=1.5
Mmp7
pv=0.02 fc=0.53
Cd248_predicted
pv=0.02 fc=0.66
Spock1
pv=0.02 fc=1.57
Cd36
pv=0.02 fc=0.56
Tgfbi
pv=0.02 fc=0.72
Dab2
pv=0.02 fc=0.67
Sirt2
pv=0.02 fc=1.6
LOC363443
pv=0.02 fc=1.34
Klk6
pv=0.02 fc=1.88
Phlda3
pv=0.02 fc=1.37

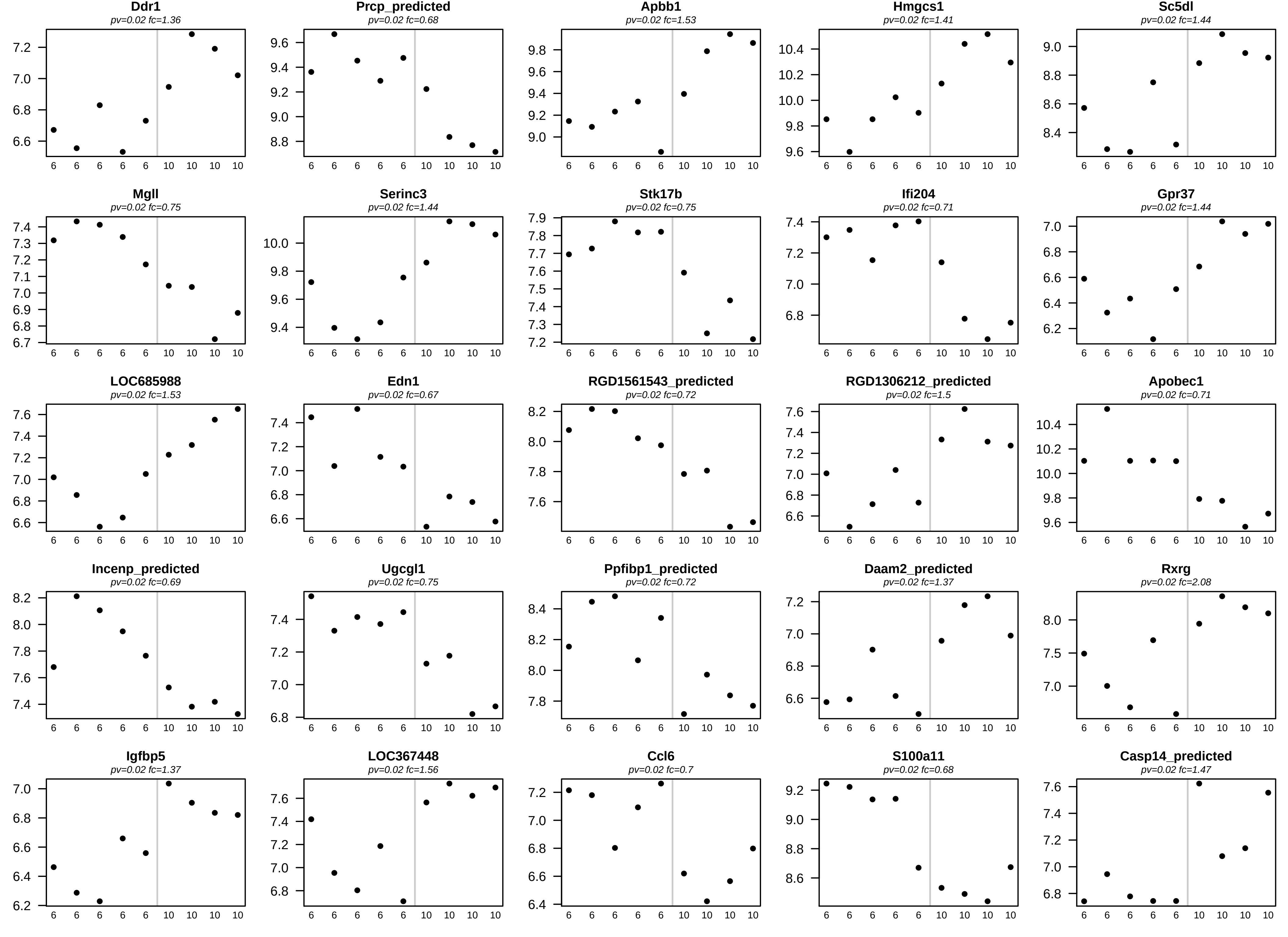
Ddr1
pv=0.02 fc=1.36
Prcp_predicted
pv=0.02 fc=0.68
Apbb1
pv=0.02 fc=1.53
Hmgcs1
pv=0.02 fc=1.41
Sc5dl
pv=0.02 fc=1.44
Mgll
pv=0.02 fc=0.75
Serinc3
pv=0.02 fc=1.44
Stk17b
pv=0.02 fc=0.75
Ifi204
pv=0.02 fc=0.71
Gpr37
pv=0.02 fc=1.44
LOC685988
pv=0.02 fc=1.53
Edn1
pv=0.02 fc=0.67
RGD1561543_predicted
pv=0.02 fc=0.72
RGD1306212_predicted
pv=0.02 fc=1.5
Apobec1
pv=0.02 fc=0.71
Incenp_predicted
pv=0.02 fc=0.69
Ugcgl1
pv=0.02 fc=0.75
Ppfibp1_predicted
pv=0.02 fc=0.72
Daam2_predicted
pv=0.02 fc=1.37
Rxrg
pv=0.02 fc=2.08
Igfbp5
pv=0.02 fc=1.37
LOC367448
pv=0.02 fc=1.56
Ccl6
pv=0.02 fc=0.7
S100a11
pv=0.02 fc=0.68
Casp14_predicted
pv=0.02 fc=1.47

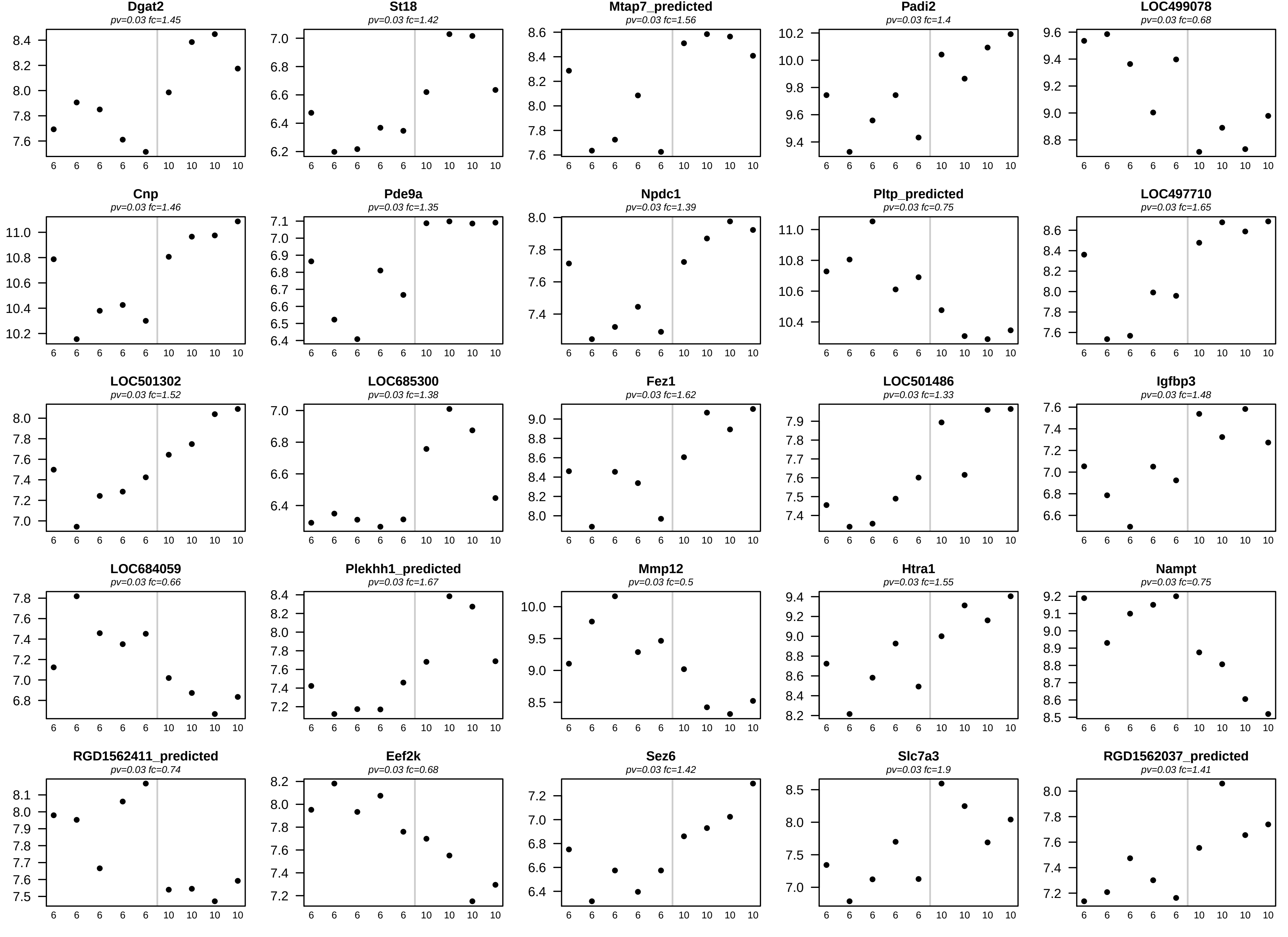
Dgat2
pv=0.03 fc=1.45
St18
pv=0.03 fc=1.42
Mtap7_predicted
pv=0.03 fc=1.56
Padi2
pv=0.03 fc=1.4
LOC499078
pv=0.03 fc=0.68
Cnp
pv=0.03 fc=1.46
Pde9a
pv=0.03 fc=1.35
Npdc1
pv=0.03 fc=1.39
Pltp_predicted
pv=0.03 fc=0.75
LOC497710
pv=0.03 fc=1.65
LOC501302
pv=0.03 fc=1.52
LOC685300
pv=0.03 fc=1.38
Fez1
pv=0.03 fc=1.62
LOC501486
pv=0.03 fc=1.33
Igfbp3
pv=0.03 fc=1.48
LOC684059
pv=0.03 fc=0.66
Plekhh1_predicted
pv=0.03 fc=1.67
Mmp12
pv=0.03 fc=0.5
Htra1
pv=0.03 fc=1.55
Nampt
pv=0.03 fc=0.75
RGD1562411_predicted
pv=0.03 fc=0.74
Eef2k
pv=0.03 fc=0.68
Sez6
pv=0.03 fc=1.42
Slc7a3
pv=0.03 fc=1.9
RGD1562037_predicted
pv=0.03 fc=1.41

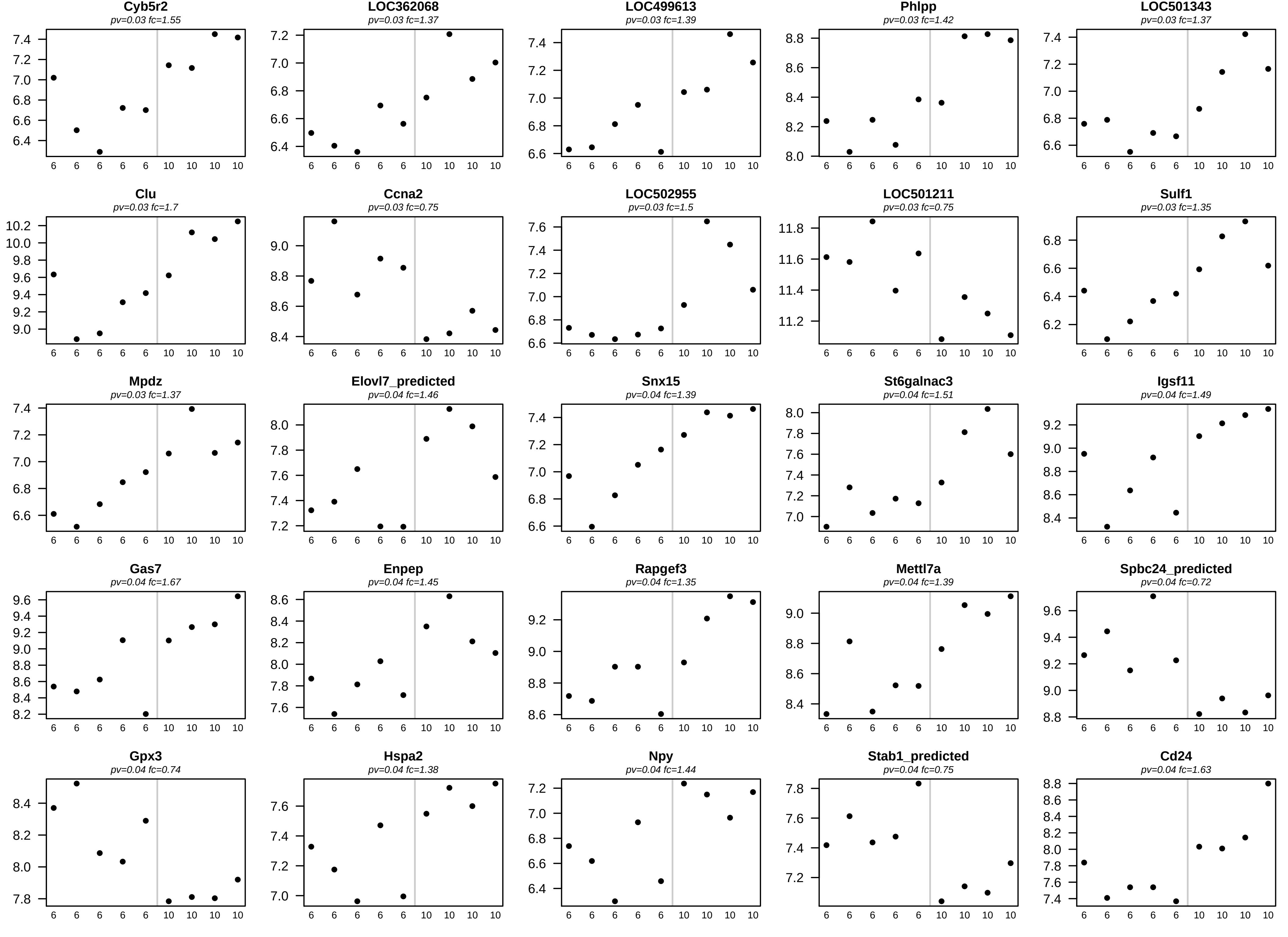

Cyb5r2
pv=0.03 fc=1.55
LOC362068
pv=0.03 fc=1.37
LOC499613
pv=0.03 fc=1.39
Phlpp
pv=0.03 fc=1.42
LOC501343
pv=0.03 fc=1.37
Clu
pv=0.03 fc=1.7
Ccna2
pv=0.03 fc=0.75
LOC502955
pv=0.03 fc=1.5
LOC501211
pv=0.03 fc=0.75
Sulf1
pv=0.03 fc=1.35
Mpdz
pv=0.03 fc=1.37
Elovl7_predicted
pv=0.04 fc=1.46
Snx15
pv=0.04 fc=1.39
St6galnac3
pv=0.04 fc=1.51
Igsf11
pv=0.04 fc=1.49
Gas7
pv=0.04 fc=1.67
Enpep
pv=0.04 fc=1.45
Rapgef3
pv=0.04 fc=1.35
Mettl7a
pv=0.04 fc=1.39
Spbc24_predicted
pv=0.04 fc=0.72
Gpx3
pv=0.04 fc=0.74
Hspa2
pv=0.04 fc=1.38
Npy
pv=0.04 fc=1.44
Stab1_predicted
pv=0.04 fc=0.75
Cd24
pv=0.04 fc=1.63

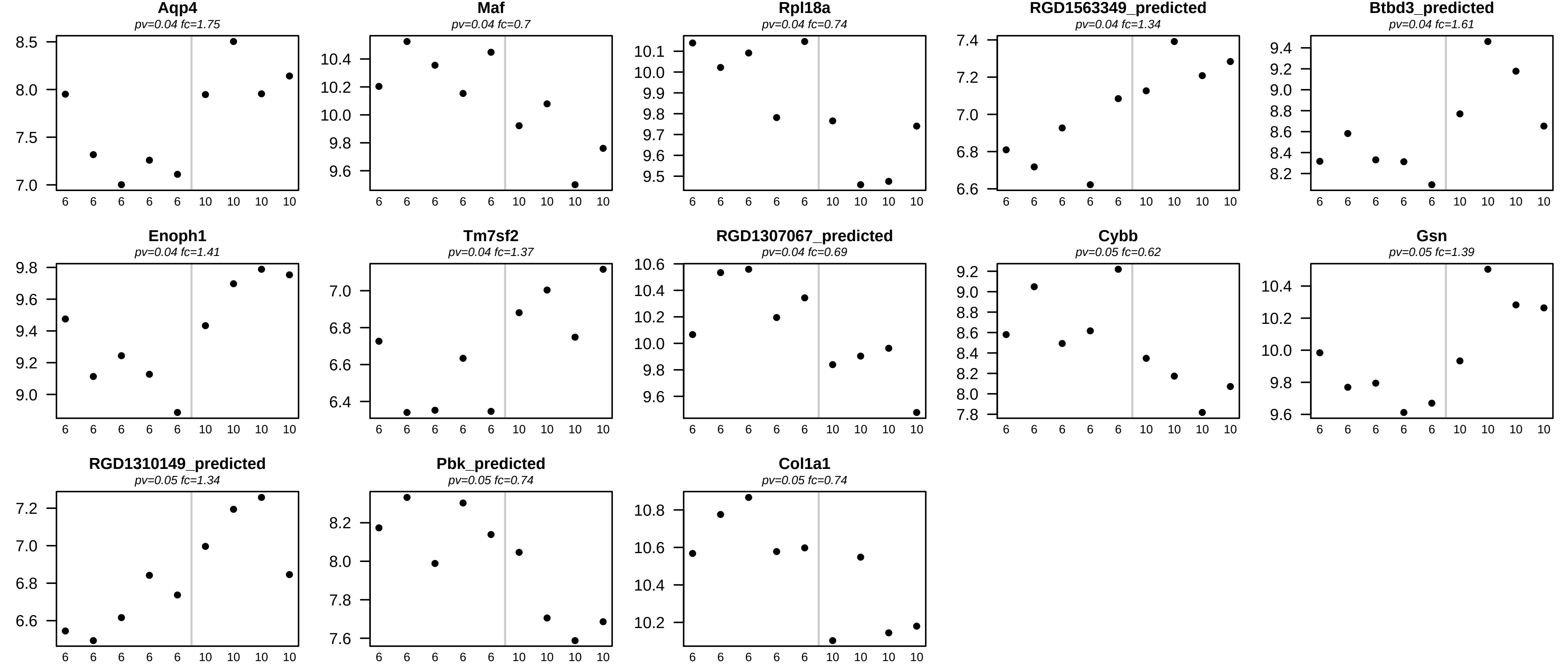
Aqp4
pv=0.04 fc=1.75
Maf
pv=0.04 fc=0.7
Rpl18a
pv=0.04 fc=0.74
RGD1563349_predicted
pv=0.04 fc=1.34
Btbd3_predicted
pv=0.04 fc=1.61
Enoph1
pv=0.04 fc=1.41
Tm7sf2
pv=0.04 fc=1.37
RGD1307067_predicted
pv=0.04 fc=0.69
Cybb
pv=0.05 fc=0.62
Gsn
pv=0.05 fc=1.39
RGD1310149_predicted
pv=0.05 fc=1.34
Pbk_predicted
pv=0.05 fc=0.74
Col1a1
pv=0.05 fc=0.74
6 6 6 6 6 10 10 10 10
