# Supplementary material for: Injury-induced perivascular niche supports alternative differentiation of adult rodent CNS progenitor cells
Source: eLife. 2018 Sep 17;7:e30325. doi: 10.7554/eLife.30325 (PMC6141235; doi:10.7554/eLife.30325)
Supplement: Figure 2—source data 2. — Dots represent level of expression for individual animal sample. [file elife-30325-fig2-data2.pdf]

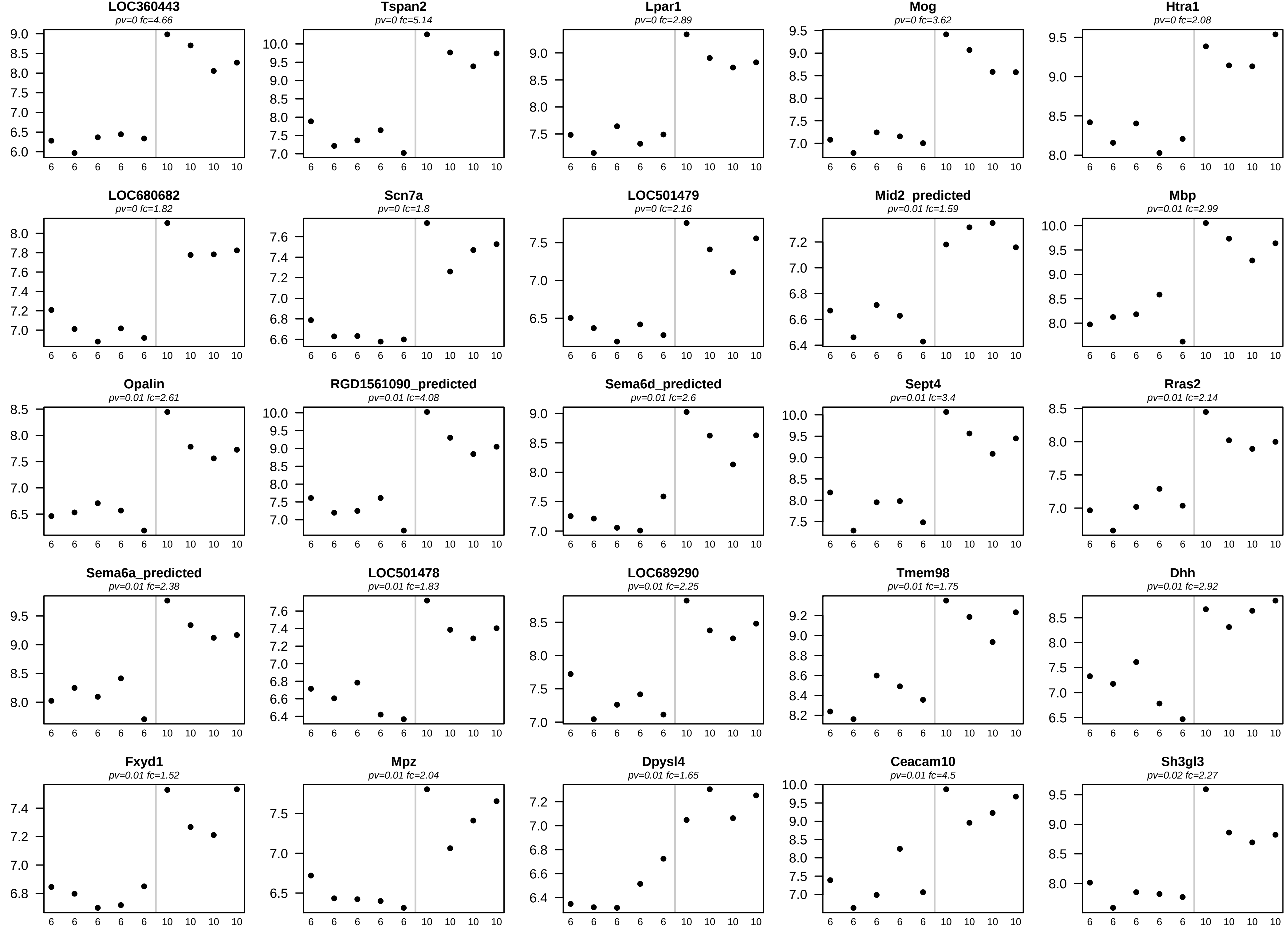
LOC360443
pv=0 fc=4.66
Tspan2
pv=0 fc=5.14
Lpar1
pv=0 fc=2.89
Mog
pv=0 fc=3.62
Htra1
pv=0 fc=2.08
LOC680682
pv=0 fc=1.82
Scn7a
pv=0 fc=1.8
LOC501479
pv=0 fc=2.16
Mid2_predicted
pv=0.01 fc=1.59
Mbp
pv=0.01 fc=2.99
Opalin
pv=0.01 fc=2.61
RGD1561090_predicted
pv=0.01 fc=4.08
Sema6d_predicted
pv=0.01 fc=2.6
Sept4
pv=0.01 fc=3.4
Rras2
pv=0.01 fc=2.14
Sema6a_predicted
pv=0.01 fc=2.38
LOC501478
pv=0.01 fc=1.83
LOC689290
pv=0.01 fc=2.25
Tmem98
pv=0.01 fc=1.75
Dhh
pv=0.01 fc=2.92
Fxyd1
pv=0.01 fc=1.52
Mpz
pv=0.01 fc=2.04
Dpysl4
pv=0.01 fc=1.65
Ceacam10
pv=0.01 fc=4.5
Sh3gl3
pv=0.02 fc=2.27
6 6 6 6 6 10 10 10 10

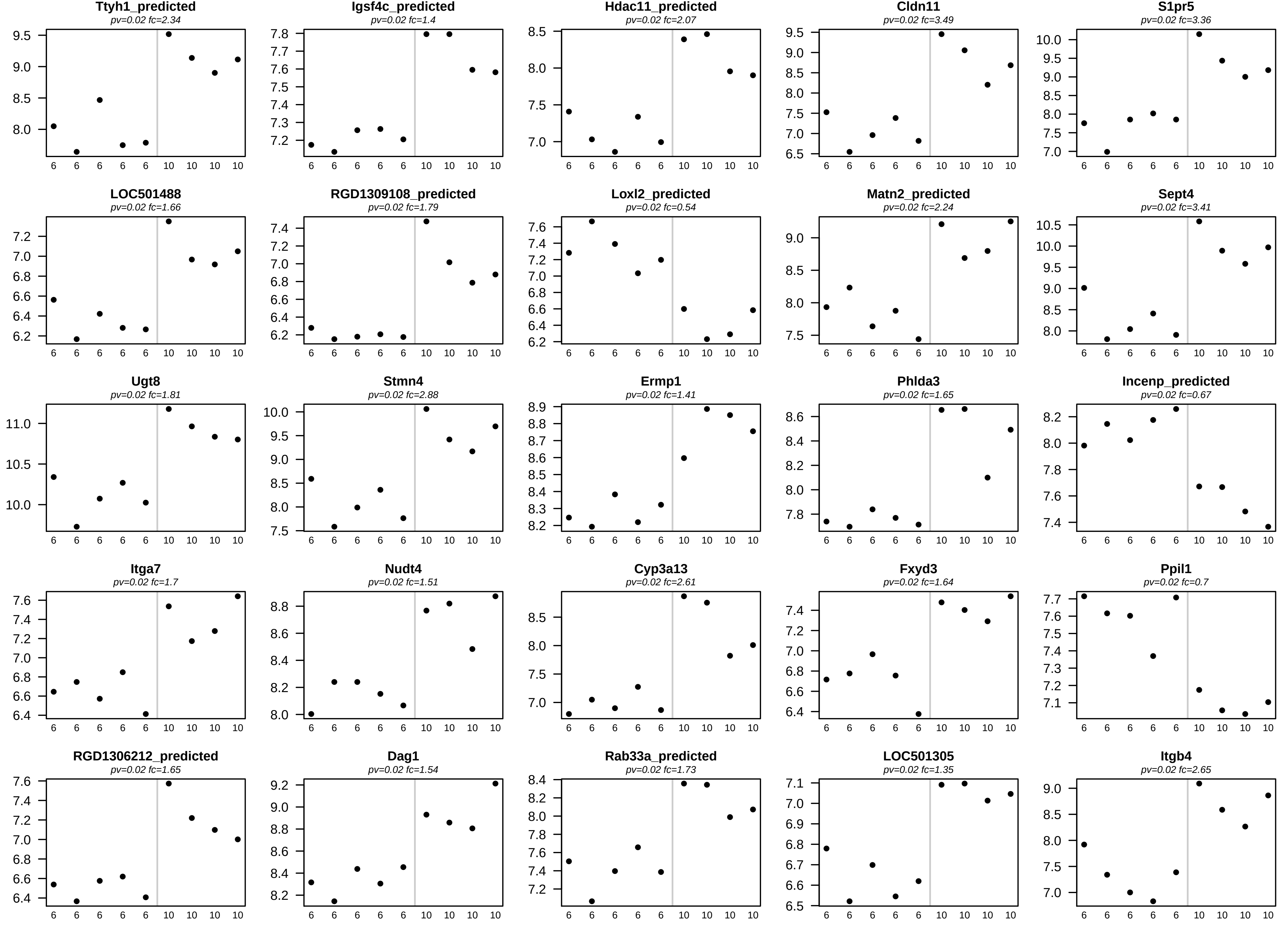
Ttyh1_predicted
pv=0.02 fc=2.34
Igsf4c_predicted
pv=0.02 fc=1.4
Hdac11_predicted
pv=0.02 fc=2.07
Cldn11
pv=0.02 fc=3.49
S1pr5
pv=0.02 fc=3.36
LOC501488
pv=0.02 fc=1.66
RGD1309108_predicted
pv=0.02 fc=1.79
Loxl2_predicted
pv=0.02 fc=0.54
Matn2_predicted
pv=0.02 fc=2.24
Sept4
pv=0.02 fc=3.41
Ugt8
pv=0.02 fc=1.81
Stmn4
pv=0.02 fc=2.88
Ermp1
pv=0.02 fc=1.41
Phlda3
pv=0.02 fc=1.65
Incenp_predicted
pv=0.02 fc=0.67
Itga7
pv=0.02 fc=1.7
Nudt4
pv=0.02 fc=1.51
Cyp3a13
pv=0.02 fc=2.61
Fxyd3
pv=0.02 fc=1.64
Ppil1
pv=0.02 fc=0.7
RGD1306212_predicted
pv=0.02 fc=1.65
Dag1
pv=0.02 fc=1.54
Rab33a_predicted
pv=0.02 fc=1.73
LOC501305
pv=0.02 fc=1.35
Itgb4
pv=0.02 fc=2.65

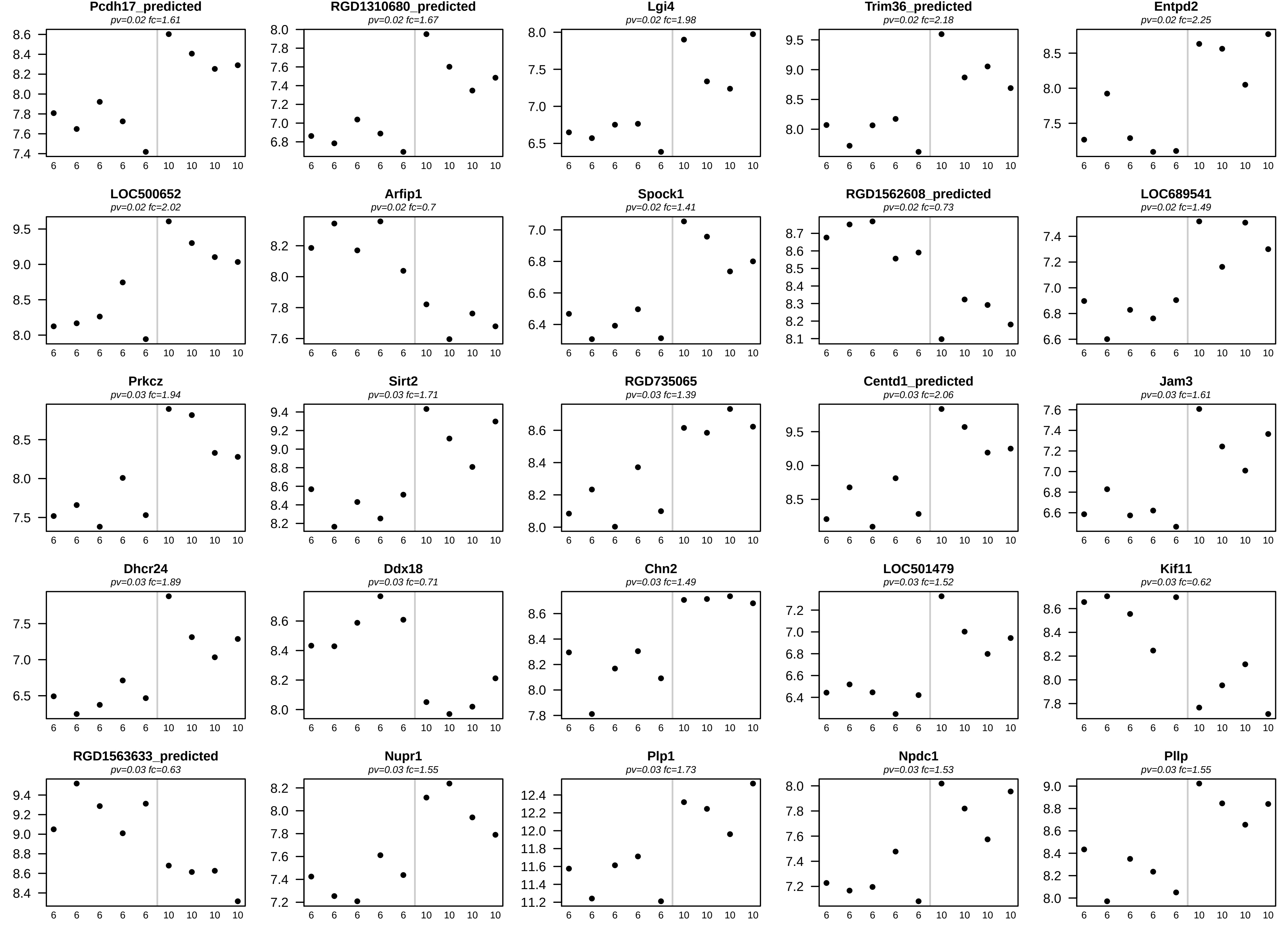

Pcdh17_predicted
pv=0.02 fc=1.61
RGD1310680_predicted
pv=0.02 fc=1.67
Lgi4
pv=0.02 fc=1.98
Trim36_predicted
pv=0.02 fc=2.18
Entpd2
pv=0.02 fc=2.25
LOC500652
pv=0.02 fc=2.02
Arfip1
pv=0.02 fc=0.7
Spock1
pv=0.02 fc=1.41
RGD1562608_predicted
pv=0.02 fc=0.73
LOC689541
pv=0.02 fc=1.49
Prkcz
pv=0.03 fc=1.94
Sirt2
pv=0.03 fc=1.71
RGD735065
pv=0.03 fc=1.39
Centd1_predicted
pv=0.03 fc=2.06
Jam3
pv=0.03 fc=1.61
Dhcr24
pv=0.03 fc=1.89
Ddx18
pv=0.03 fc=0.71
Chn2
pv=0.03 fc=1.49
LOC501479
pv=0.03 fc=1.52
Kif11
pv=0.03 fc=0.62
RGD1563633_predicted
pv=0.03 fc=0.63
Nupr1
pv=0.03 fc=1.55
Plp1
pv=0.03 fc=1.73
Npdc1
pv=0.03 fc=1.53
Pllp
pv=0.03 fc=1.55

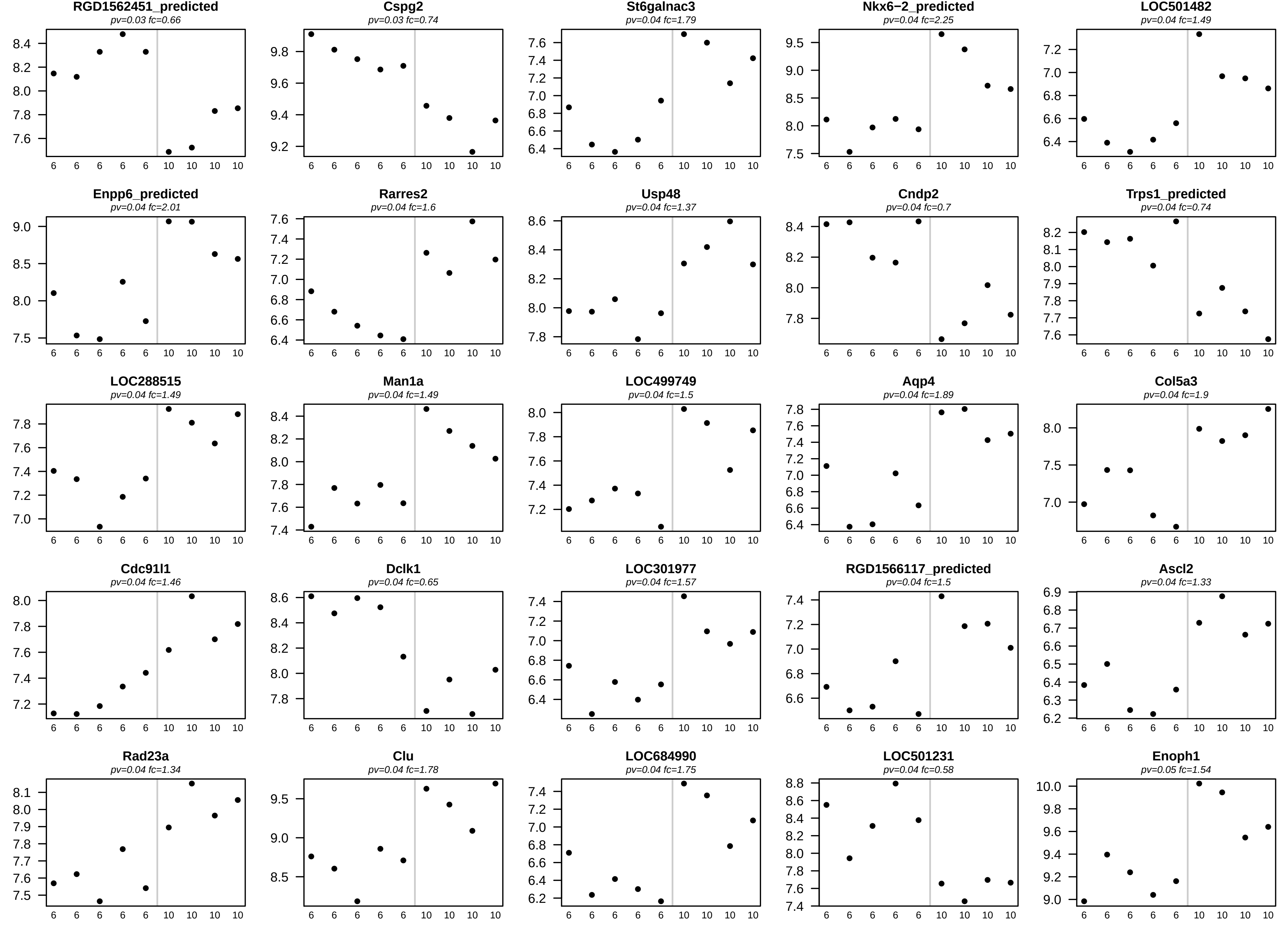
RGD1562451_predicted
pv=0.03 fc=0.66
Cspg2
pv=0.03 fc=0.74
St6galnac3
pv=0.04 fc=1.79
Nkx6−2_predicted
pv=0.04 fc=2.25
LOC501482
pv=0.04 fc=1.49
Enpp6_predicted
pv=0.04 fc=2.01
Rarres2
pv=0.04 fc=1.6
Usp48
pv=0.04 fc=1.37
Cndp2
pv=0.04 fc=0.7
Trps1_predicted
pv=0.04 fc=0.74
LOC288515
pv=0.04 fc=1.49
Man1a
pv=0.04 fc=1.49
LOC499749
pv=0.04 fc=1.5
Aqp4
pv=0.04 fc=1.89
Col5a3
pv=0.04 fc=1.9
Cdc91l1
pv=0.04 fc=1.46
Dclk1
pv=0.04 fc=0.65
LOC301977
pv=0.04 fc=1.57
RGD1566117_predicted
pv=0.04 fc=1.5
Ascl2
pv=0.04 fc=1.33
Rad23a
pv=0.04 fc=1.34
Clu
pv=0.04 fc=1.78
LOC684990
pv=0.04 fc=1.75
LOC501231
pv=0.04 fc=0.58
Enoph1
pv=0.05 fc=1.54

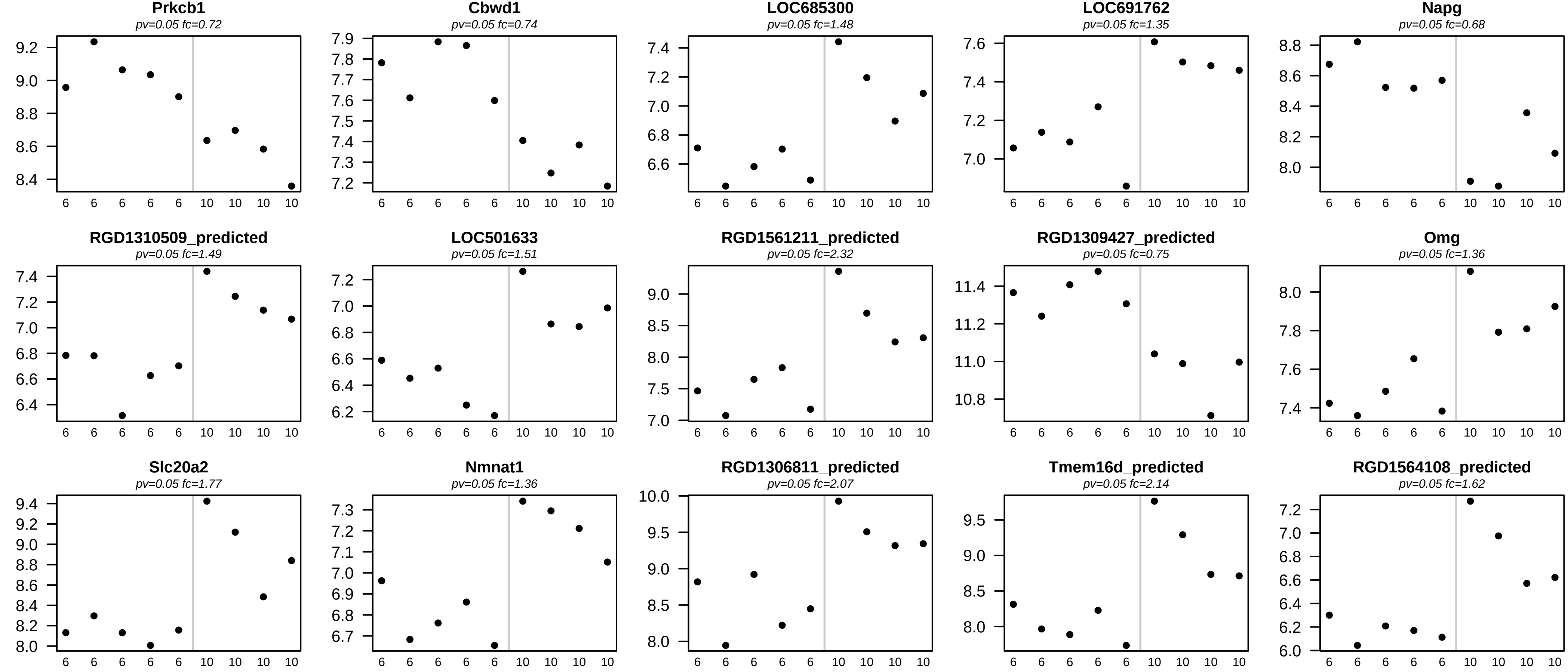
Prkcb1
pv=0.05 fc=0.72
Cbwd1
pv=0.05 fc=0.74
LOC685300
pv=0.05 fc=1.48
LOC691762
pv=0.05 fc=1.35
Napg
pv=0.05 fc=0.68
RGD1310509_predicted
pv=0.05 fc=1.49
LOC501633
pv=0.05 fc=1.51
RGD1561211_predicted
pv=0.05 fc=2.32
RGD1309427_predicted
pv=0.05 fc=0.75
Omg
pv=0.05 fc=1.36
Slc20a2
pv=0.05 fc=1.77
Nmnat1
pv=0.05 fc=1.36
RGD1306811_predicted
pv=0.05 fc=2.07
Tmem16d_predicted
pv=0.05 fc=2.14
RGD1564108_predicted
pv=0.05 fc=1.62
