# Supplementary material for: Injury-induced perivascular niche supports alternative differentiation of adult rodent CNS progenitor cells
Source: eLife. 2018 Sep 17;7:e30325. doi: 10.7554/eLife.30325 (PMC6141235; doi:10.7554/eLife.30325)
Supplement: Figure 2—source data 3. — Dots represent level of expression for individual animal sample. [file elife-30325-fig2-data3.pdf]

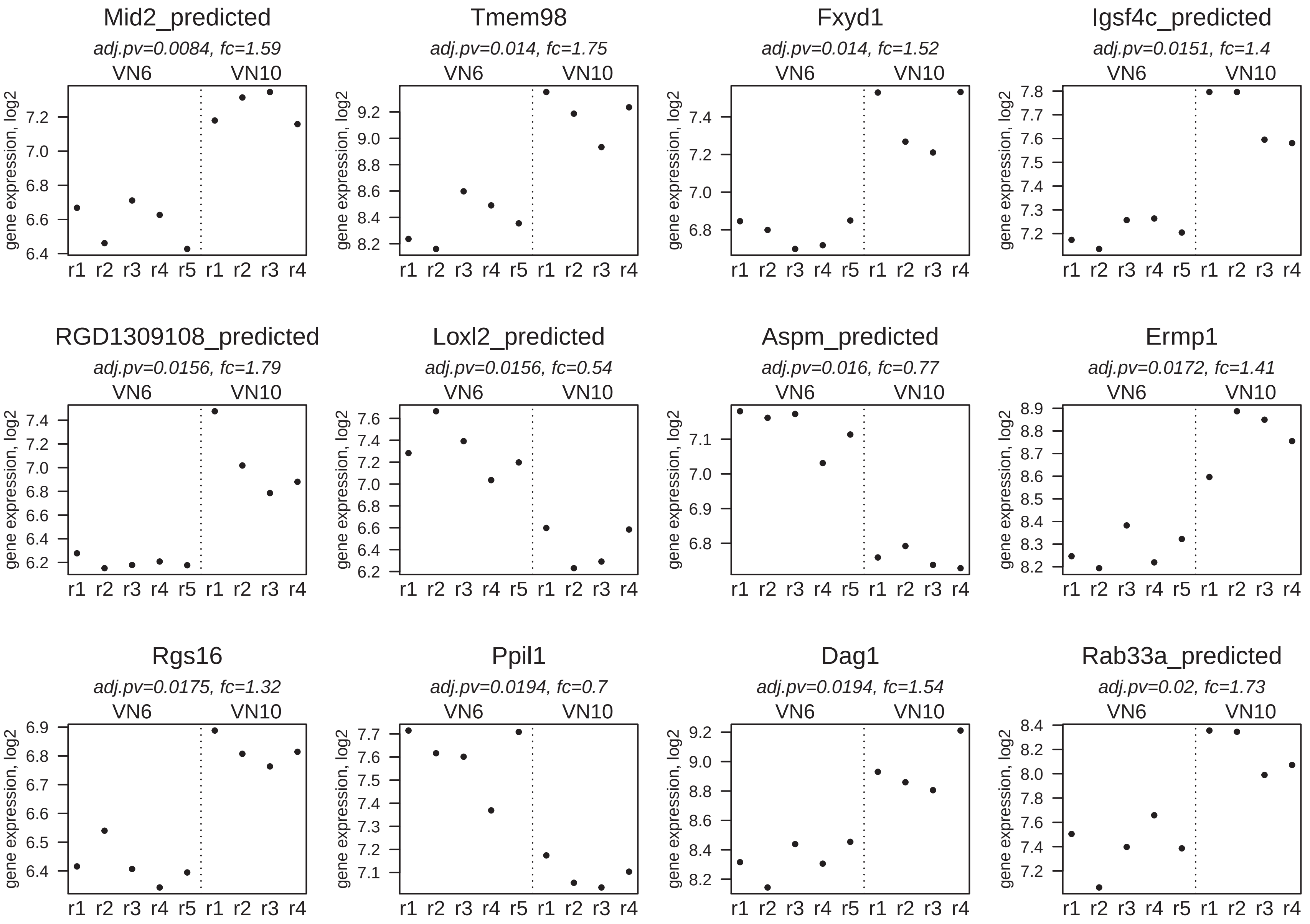
Mid2_predicted
adj.pv=0.0084, fc=1.59
Tmem98
adj.pv=0.014, fc=1.75
Fxyd1
adj.pv=0.014, fc=1.52
Igsf4c_predicted
adj.pv=0.0151, fc=1.4
RGD1309108_predicted
adj.pv=0.0156, fc=1.79
Loxl2_predicted
adj.pv=0.0156, fc=0.54
Aspm_predicted
adj.pv=0.016, fc=0.77
Ermp1
adj.pv=0.0172, fc=1.41
Rgs16
adj.pv=0.0175, fc=1.32
Ppil1
adj.pv=0.0194, fc=0.7
Dag1
adj.pv=0.0194, fc=1.54
Rab33a_predicted
adj.pv=0.02, fc=1.73
VN6
VN10
gene expression, log2
r1 r2 r3 r4 r5 r1 r2 r3 r4

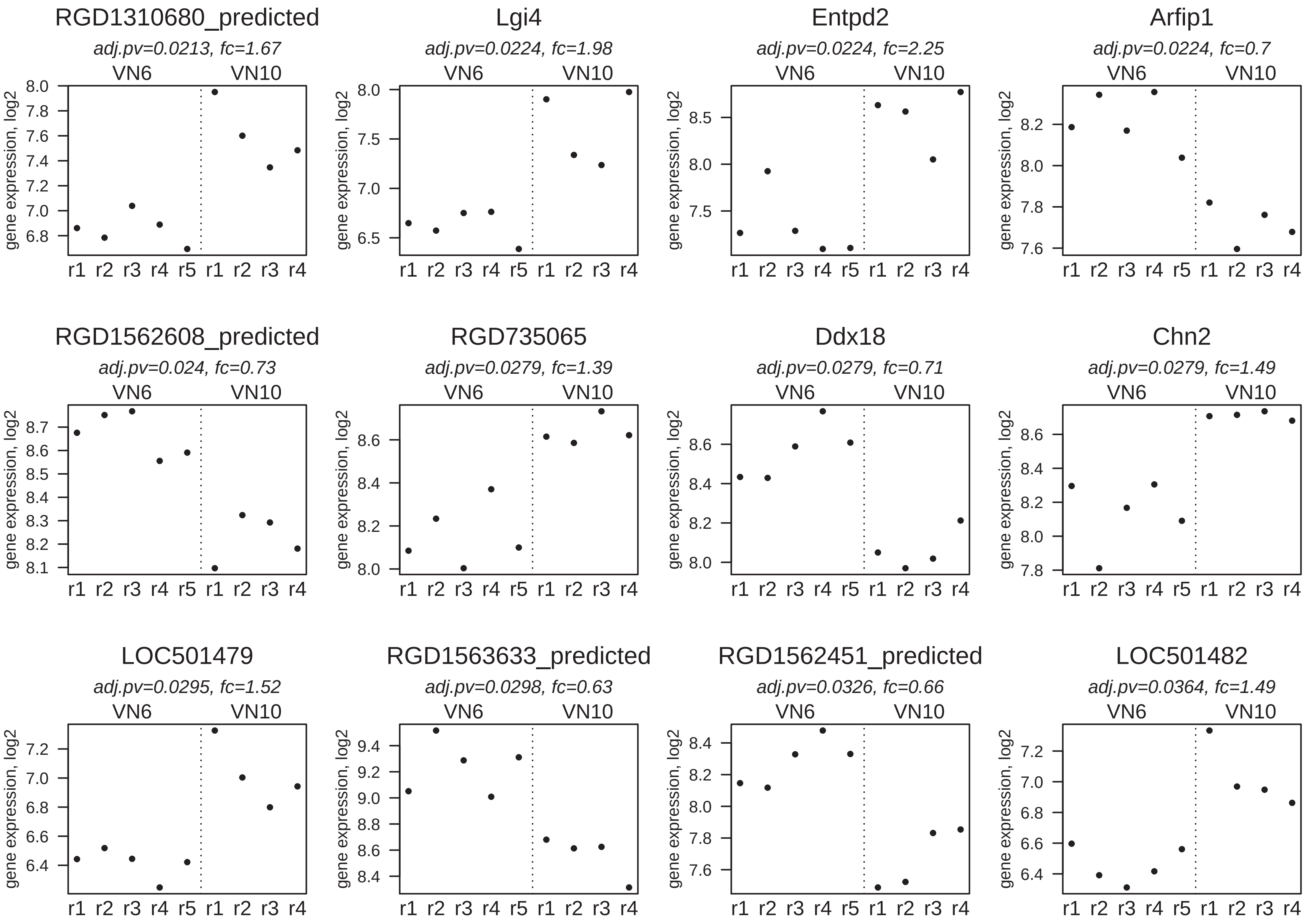

RGD1310680_predicted
adj.pv=0.0213, fc=1.67
Lgi4
adj.pv=0.0224, fc=1.98
Entpd2
adj.pv=0.0224, fc=2.25
Arfip1
adj.pv=0.0224, fc=0.7
RGD1562608_predicted
adj.pv=0.024, fc=0.73
RGD735065
adj.pv=0.0279, fc=1.39
Ddx18
adj.pv=0.0279, fc=0.71
Chn2
adj.pv=0.0279, fc=1.49
LOC501479
adj.pv=0.0295, fc=1.52
RGD1563633_predicted
adj.pv=0.0298, fc=0.63
RGD1562451_predicted
adj.pv=0.0326, fc=0.66
LOC501482
adj.pv=0.0364, fc=1.49
VN6
VN10
gene expression, log2
r1 r2 r3 r4 r5 r1 r2 r3 r4

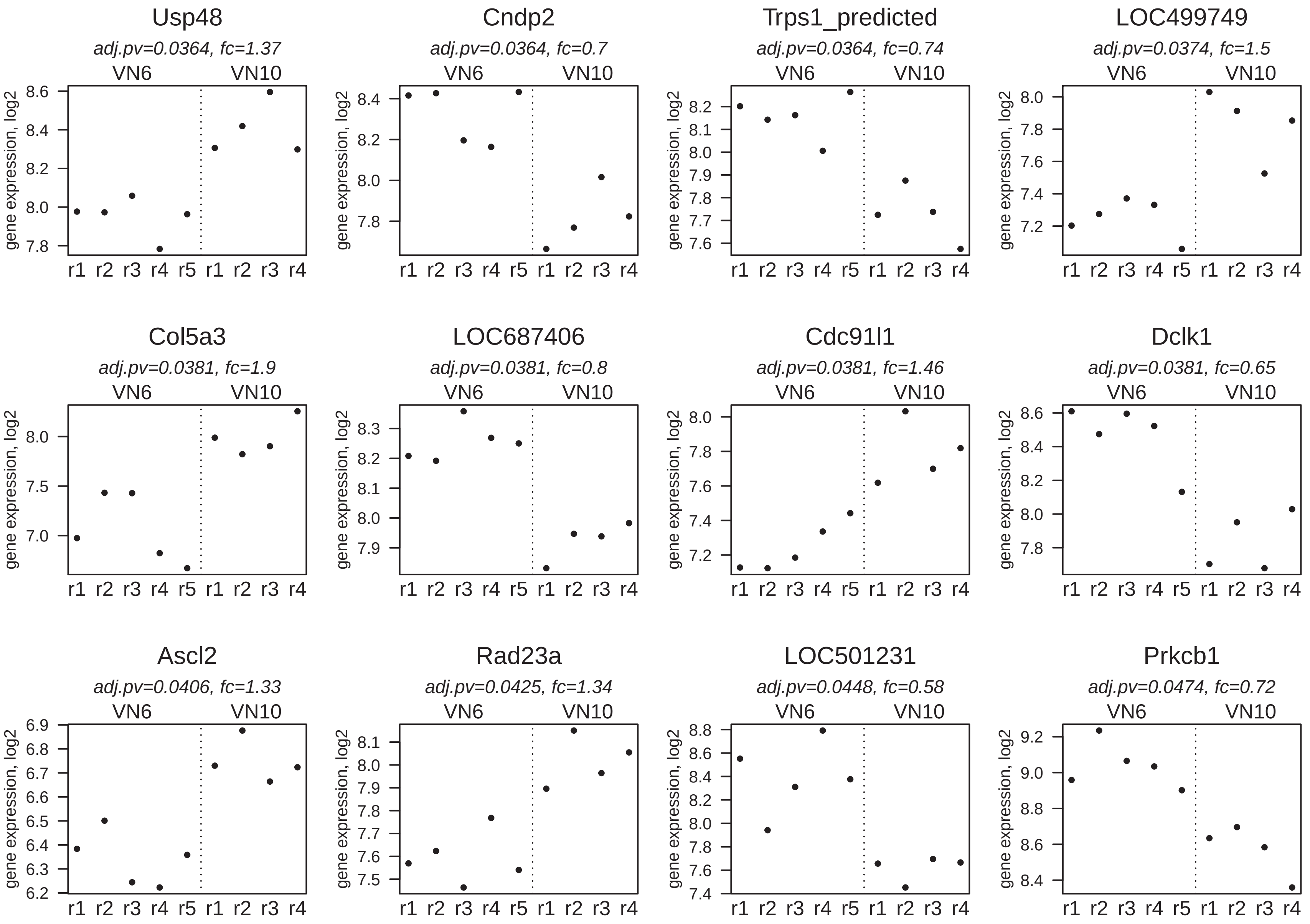

Usp48
adj.pv=0.0364, fc=1.37
Cndp2
adj.pv=0.0364, fc=0.7
Trps1_predicted
adj.pv=0.0364, fc=0.74
LOC499749
adj.pv=0.0374, fc=1.5
Col5a3
adj.pv=0.0381, fc=1.9
LOC687406
adj.pv=0.0381, fc=0.8
Cdc91l1
adj.pv=0.0381, fc=1.46
Dclk1
adj.pv=0.0381, fc=0.65
Ascl2
adj.pv=0.0406, fc=1.33
Rad23a
adj.pv=0.0425, fc=1.34
LOC501231
adj.pv=0.0448, fc=0.58
Prkcb1
adj.pv=0.0474, fc=0.72
VN6
VN10
gene expression, log2
r1 r2 r3 r4 r5 r1 r2 r3 r4

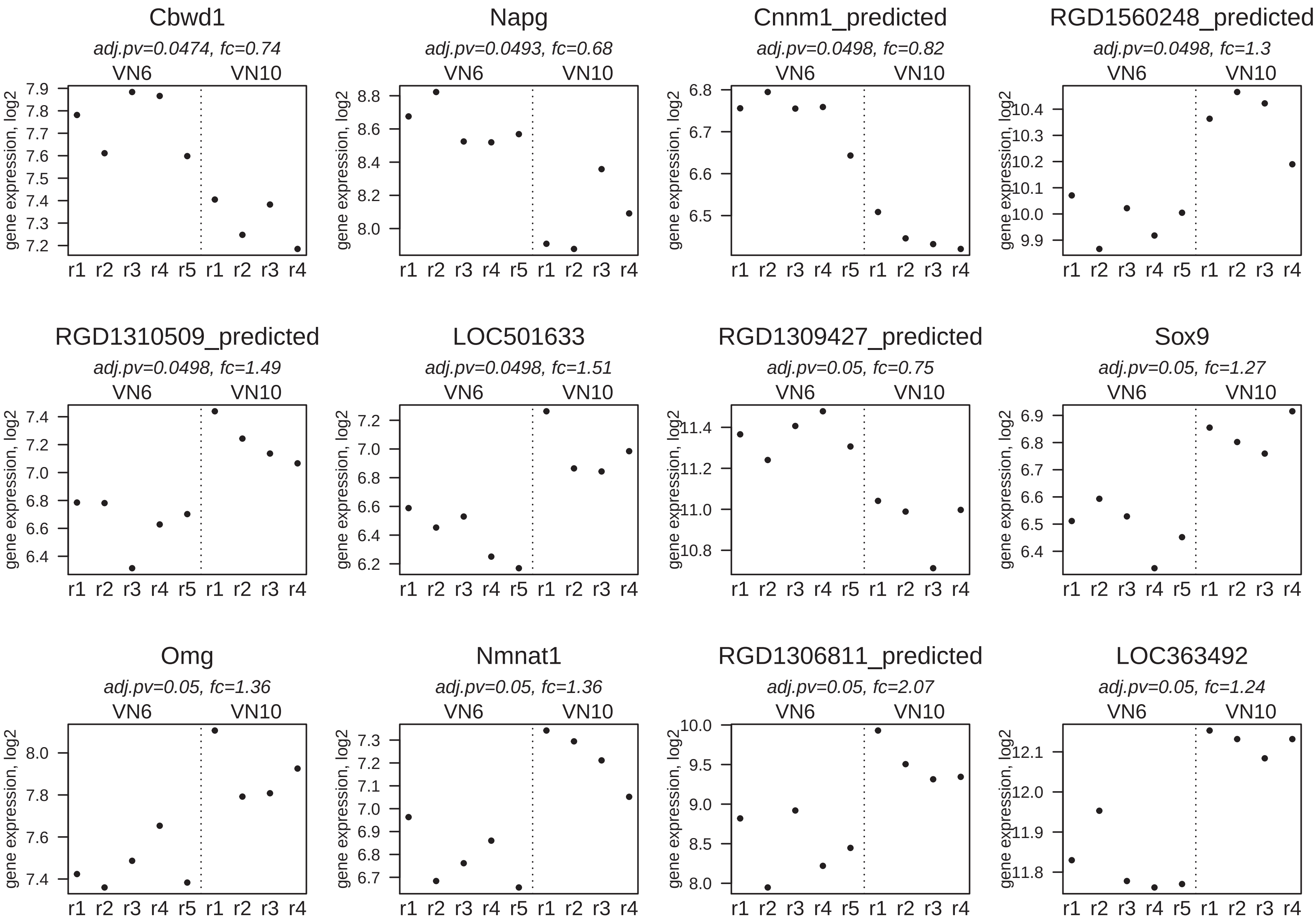
Cbwd1
adj.pv=0.0474, fc=0.74
Napg
adj.pv=0.0493, fc=0.68
Cnnm1_predicted
adj.pv=0.0498, fc=0.82
RGD1560248_predicted
adj.pv=0.0498, fc=1.3
RGD1310509_predicted
adj.pv=0.0498, fc=1.49
LOC501633
adj.pv=0.0498, fc=1.51
RGD1309427_predicted
adj.pv=0.05, fc=0.75
Sox9
adj.pv=0.05, fc=1.27
Omg
adj.pv=0.05, fc=1.36
Nmnat1
adj.pv=0.05, fc=1.36
RGD1306811_predicted
adj.pv=0.05, fc=2.07
LOC363492
adj.pv=0.05, fc=1.24
VN6
VN10
gene expression, log2
r1 r2 r3 r4 r5 r1 r2 r3 r4
