# Supplementary material for: Injury-induced perivascular niche supports alternative differentiation of adult rodent CNS progenitor cells
Source: eLife. 2018 Sep 17;7:e30325. doi: 10.7554/eLife.30325 (PMC6141235; doi:10.7554/eLife.30325)
Supplement: Transparent reporting form [file elife-30325-transrepform.docx]

***eLife’s* transparent reporting form**

We encourage authors to provide detailed information *within their submission* to facilitate the interpretation and replication of experiments. Authors can upload supporting documentation to indicate the use of appropriate reporting guidelines for health-related research (see [EQUATOR Network](http://www.equator-network.org/%20)), life science research (see the [BioSharing Information Resource](https://biosharing.org/)), or the [ARRIVE guidelines](http://www.plosbiology.org/article/info:doi/10.1371/journal.pbio.1000412) for reporting work involving animal research. Where applicable, authors should refer to any relevant reporting standards documents in this form.

If you have any questions, please consult our Journal Policies and/or contact us: [editorial@elifesciences.org](mailto:editorial@elifesciences.org).

**Sample-size estimation**

- You should state whether an appropriate sample size was computed when the study was being designed
- You should state the statistical method of sample size computation and any required assumptions
- If no explicit power analysis was used, you should describe how you decided what sample (replicate) size (number) to use

Please outline where this information can be found within the submission (e.g., sections or figure legends), or explain why this information doesn’t apply to your submission:

We did not perform statistical analyses to compute the sample size in our experiments. To test our assumptions, we performed, however, post hoc sample size calculation on previously obtained means and sd with the same experimental model taking alpha Prob(reject H0 when H0 is true) as 0.05 and Power: Prob(reject H0 when H1 is true) as 0.8 based on G.W. Snedecor & W.G. Cochran Statistical Methods (7th ed., p. 104) which showed the sample size needed was less than 6.

**Replicates**

- You should report how often each experiment was performed
- You should include a definition of biological versus technical replication
- The data obtained should be provided and sufficient information should be provided to indicate the number of independent biological and/or technical replicates
- If you encountered any outliers, you should describe how these were handled
- Criteria for exclusion/inclusion of data should be clearly stated
- High-throughput sequence data should be uploaded before submission, with a private link for reviewers provided (these are available from both GEO and ArrayExpress)

Please outline where this information can be found within the submission (e.g., sections or figure legends), or explain why this information doesn’t apply to your submission:

We performed our experiments in following biological replicates:

1. five biological replicates for microarray analysis of gene expression (n=5 for each time point). RNA samples obtained from obtained from both lesioned tissue of each animal were pulled as VN and non-VN, separately and total of 20 hybridizations were performed, with five biological replicates per condition.

Two microarrays (derived from one animal, VN and non-VN areas) which did not accomplish our highest standards of analysis have been excluded from comparison.

The microarray dataset has been deposited in the Gene Expression Omnibus repository, and is available with the accession number GSE93645.

2. at least three biological replicates for qPCR gene expression analysis (all in triplicate technical measurements)

3. at least three biological replicates (spinal cords or sciatic nerves) were used for cellular quantification in immunofluorescence analyses (at least three tissue sections were analysed per animal)

4. three independent experiments from individual culture preparations for all *in vitro* assays were done.

**Statistical reporting**

- Statistical analysis methods should be described and justified
- Raw data should be presented in figures whenever informative to do so (typically when N per group is less than 10)
- For each experiment, you should identify the statistical tests used, exact values of N, definitions of center, methods of multiple test correction, and dispersion and precision measures (e.g., mean, median, SD, SEM, confidence intervals; and, for the major substantive results, a measure of effect size (e.g., Pearson's r, Cohen's d)
- Report exact p-values wherever possible alongside the summary statistics and 95% confidence intervals. These should be reported for all key questions and not only when the p-value is less than 0.05.

Please outline where this information can be found within the submission (e.g., sections or figure legends), or explain why this information doesn’t apply to your submission:

Statistics Prism 6.0 (GraphPad) software was used for statistical analysis except microarray analysis. Data are presented as mean ± sd.

Microarray data were exported from Illumina BeadStudio software, processed and normalized using the R/Bioconductor beadarray, lumi and limma packages. Before normalization, probes that were not detected (detection P value>0.01) were removed from further analysis. Remaining probes were mapped to gene identifiers from Ensembl database (gene_stable_id). For each gene, we computed a single average intensity profile from the profiles of all the probes mapped to it. The resulting average profile was then log2-transformed and used in statistical analysis and visualization.

Differential expression analyses were conducted using the limma Bioconductor package. False Discovery Rate (FDR) was used to adjust for multiple hypothesis testing (Benjamini, 1995). Genes with FDR < 0.05 and with at least 1.5-fold change in expression levels were found as differentially expressed. Signalling pathways described in the Kyoto Encyclopaedia of Genes and Genomes (KEGG, 2000) were tested for overrepresentation in the list of differentially expressed genes using Fisher’s exact test. Differentially expressed genes between the niches were detected and volcano plot was created.

We verified microarray data by qPCR and the gene expression was analyzed as follows: first, we normalized expression the genes of interest from each experimental condition to the level of housekeeping gene 18s RNA and b-actin separately for each sample. Second, we compared each normalized value to the average of the particular gene expression in the intact white matter (controls, n=3). Then we averaged these normalized data across all samples for each group to obtain the mean compared to control intact tissue.

To detect differences between experimental conditions Student’s t-test or non-parametric Mann-Whithney U-test were performed, as appropriate. pv<0.05 was considered to be statistically significant. Multiple group comparisons were conducted using one-way ANOVA followed by Newman-Keuls post hoc test. All in vitro assays represent three independent experiments from individual culture preparations.

Numbers of animals and cells studied, means, sd, pv, and adj.pv are stated for each experiment in the figures and figures legend.

(For large datasets, or papers with a very large number of statistical tests, you may upload a single table file with tests, Ns, etc., with reference to sections in the manuscript.)

**Group allocation**

- Indicate how samples were allocated into experimental groups (in the case of clinical studies, please specify allocation to treatment method); if randomization was used, please also state if restricted randomization was applied
- Indicate if masking was used during group allocation, data collection and/or data analysis

Please outline where this information can be found within the submission (e.g., sections or figure legends), or explain why this information doesn’t apply to your submission:

Animal were randomly assigned to experimental groups.

**Additional data files (“source data”)**

- We encourage you to upload relevant additional data files, such as numerical data that are represented as a graph in a figure, or as a summary table
- Where provided, these should be in the most useful format, and they can be uploaded as “Source data” files linked to a main figure or table
- Include model definition files including the full list of parameters used
- Include code used for data analysis (e.g., R, MatLab)
- Avoid stating that data files are “available upon request”

Please indicate the figures or tables for which source data files have been provided:

Supplementary figures 2-4, related to figure 2, provide the source data for genes differentially expressed in predefined areas of demyelinated white matter.
